# Feasibility of and barriers to thalassemia screening in migrant populations: a cross-sectional study of Myanmar and Cambodian migrants in Thailand

**DOI:** 10.1186/s12889-021-11059-2

**Published:** 2021-06-21

**Authors:** Julia Z. Xu, Wilaslak Tanongsaksakul, Thidarat Suksangpleng, Supachai Ekwattanakit, Suchada Riolueang, Marilyn J. Telen, Vip Viprakasit

**Affiliations:** 1 Department of Medicine, Duke University, Durham, USA; 2 National Heart, Lung, and Blood Institute, National Institutes of Health, 10 Center Drive, MD 20892 Bethesda, USA; 3 Department of Pediatrics, Laem Chabang Hospital, Laem Chabang, Chonburi 20230 Thailand; 4 Thalassemia Center, Faculty of Medicine Siriraj Hospital, Mahidol University, 2 Wanglang Road, Bangkoknoi, Bangkok, 10700 Thailand; 5 Department of Pediatrics, Faculty of Medicine Siriraj Hospital, Mahidol University, 2 Wanglang Road, Bangkoknoi, Bangkok, 10700 Thailand; 6 Thalassemia Center and Department of Pediatrics, Faculty of Medicine Siriraj Hospital, Mahidol University, 2 Wanglang Road, Bangkoknoi, Bangkok, 10700 Thailand

**Keywords:** Thalassemia, Hemoglobin E, Migrants, Awareness, KAP survey, Cross-cultural comparison, Epidemiology, Genetic testing, Thailand, Southeast Asia

## Abstract

**Background:**

Thalassemia, an inherited hemoglobin disorder, has become a global public health problem due to population migration. Evidence-based strategies for thalassemia prevention in migrants are lacking. We characterized barriers to thalassemia screening and the burden of thalassemia in migrant workers in Thailand.

**Methods:**

Multilingual demographic and KAP surveys were completed by 197 Thai, 119 Myanmar, and 176 Cambodian adults residing in Thailand. Thalassemia awareness, socio-demographic predictors, and knowledge and attitude scores were compared between migrant and Thai subjects. Comprehensive thalassemia testing was performed for migrants.

**Results:**

Migrants had extremely poor thalassemia awareness (4.1%) compared to Thai subjects (79.6%) and had lower thalassemia knowledge scores but similar attitude scores. Surveys identified differing sociodemographic factors predicting awareness in Thai and migrant subjects, as well as key misconceptions likely to hinder thalassemia screening uptake. Nearly all migrants consented to thalassemia testing. We identified abnormal hemoglobin profiles in 52.7% of migrants and a higher projected rate of severe thalassemia births in migrants.

**Conclusions:**

The high burden of thalassemia and tremendous knowledge gap in migrants needs urgent attention. Thalassemia screening was feasible and acceptable in our migrant population. Sociocultural and structural barriers merit further attention when designing thalassemia screening and prevention policies for migrants in Thailand and globally.

**Supplementary Information:**

The online version contains supplementary material available at 10.1186/s12889-021-11059-2.

## Background

Thalassemia syndromes, caused by impaired α- and/or β-globin chain synthesis, together with sickle cell disease represent the most common human Mendelian disorders. There are an estimated ~ 56,000 annual births globally with severe thalassemia (hemoglobin [Hb] Bart’s hydrops fetalis, Hb H disease, β-thalassemia major, and Hb E/β-thalassemia) [1], though the burden of all clinically significant thalassemia disease likely far exceeds this estimate. Due to increasing international migration, the epidemiology of hemoglobin disorders is changing rapidly, and the resulting new challenges posed to health services have been recognized in Europe and the United States [2–4]. Southeast Asia (SEA) is severely affected by thalassemia syndromes [5], with high frequencies of both α- and β-thalassemia, including Hb E (β26 Glu➔Lys), a structural variant that reduces β-globin expression (Table 1).

**Table 1:** Carrier rates (in %) for common hemoglobin disorders in Southeast Asia^a^

Country	α^**0**^-thalassemia (%)	α^**+**^-thalassemia (%)	β-thalassemia (%)	Hemoglobin E (%)
Brunei [6, 7]	0	4.3	2–22.7	0–3.7
Cambodia	1	15.5	2.8	10–54
Indonesia	< 1	3–20	3	1–33
Laos [8, 9]	8.7–13.9	11–17.7	3.5–5	22.9– > 30
Malaysia	4.5	16	4.5	1–3
Myanmar	NA	10	1–5.3	4–48
Philippines	5	2.2	1	NA
Singapore	2–3	1–3	0.93	0.64
Thailand	2.2–9	8–30	1–3	10–50
Vietnam	NA	3.5	4	10–20

“%” refers to the percentage of individuals affected (i.e. % carriers) within the population. *NA* Not available

^a^ Adapted from Viprakasit et al., 2009 [10].

Thailand has one of the highest burdens of thalassemia in the world, with ~ 15 million carriers and 50,000 pregnancies at risk annually for severe thalassemia [11]. Thailand is also one of the few SEA countries with effective severe thalassemia prevention and control policies, focusing on public education, prenatal screening, and prenatal diagnosis. This has led to a decreased incidence of severe thalassemia syndromes, including Hb Bart’s hydrops fetalis [11]. Concurrently, Thailand’s relatively prosperous and stable economy has encouraged increasing migration from neighboring countries, with 2.4 million migrant workers from Myanmar, Cambodia, and Laos [12, 13]. These three neighboring countries are also estimated to have a high prevalence of thalassemia and Hb E (Table 1). However, they do not have national thalassemia education, screening, or counseling programs in place, and data on the incidence of severe thalassemia syndromes are limited due to lack of routine screening [14]. With increasing intra-regional migration, the lack of access to thalassemia-related services among migrant populations in Thailand not only hinders early diagnosis and treatment but also potentially undermines the Thai thalassemia program’s long-term success. Anecdotally, a rise in the number of births affected by severe thalassemia among migrant couples has been observed (V. Viprakasit, personal communication, November 1, 2017).

Addressing the issue of thalassemia in migrant populations is therefore of utmost importance but fraught with challenges. Migrant workers are often low-skilled, enter through irregular channels, and have limited or no access to healthcare. There are also large populations of asylum seekers and refugees from Myanmar and other neighboring countries who have crossed the border into Thailand irregularly and reside in refugee camps and border towns. The lack of epidemiological data regarding disease burden in different migrant populations hinders policymakers’ abilities to weigh the social benefit and economic cost of implementing thalassemia programs. Additionally, knowledge gaps and cultural differences are known barriers to the uptake of screening and the effectiveness of thalassemia prevention efforts [15–17], yet few studies have examined knowledge, attitudes, and health-seeking behaviors among SEA migrants. How best to engage migrants in thalassemia education and screening and whether screening is feasible and acceptable to migrant populations must be explored.

We developed demographic and knowledge, attitudes, and practices (KAP) surveys to identify potential barriers to thalassemia screening among Myanmar and Cambodian migrant workers residing in Chonburi, an industrial province within the Eastern Economic Corredor (EEC), a major growing economic region in Thailand and home to one of the largest migrant worker populations in the country. We hypothesized that migrant subjects would have less awareness of thalassemia compared to Thai subjects and that knowledge would correlate with attitudes towards thalassemia screening and prevention. Furthermore, we performed comprehensive thalassemia testing to demonstrate the disease burden and assess the feasibility and acceptability of thalassemia screening in migrants. Novel insights from this study may inform regional migrant health policy and improve thalassemia education, prevention, and care for migrant populations globally.

## Methods

### Study setting and population

**Fig. 1:**
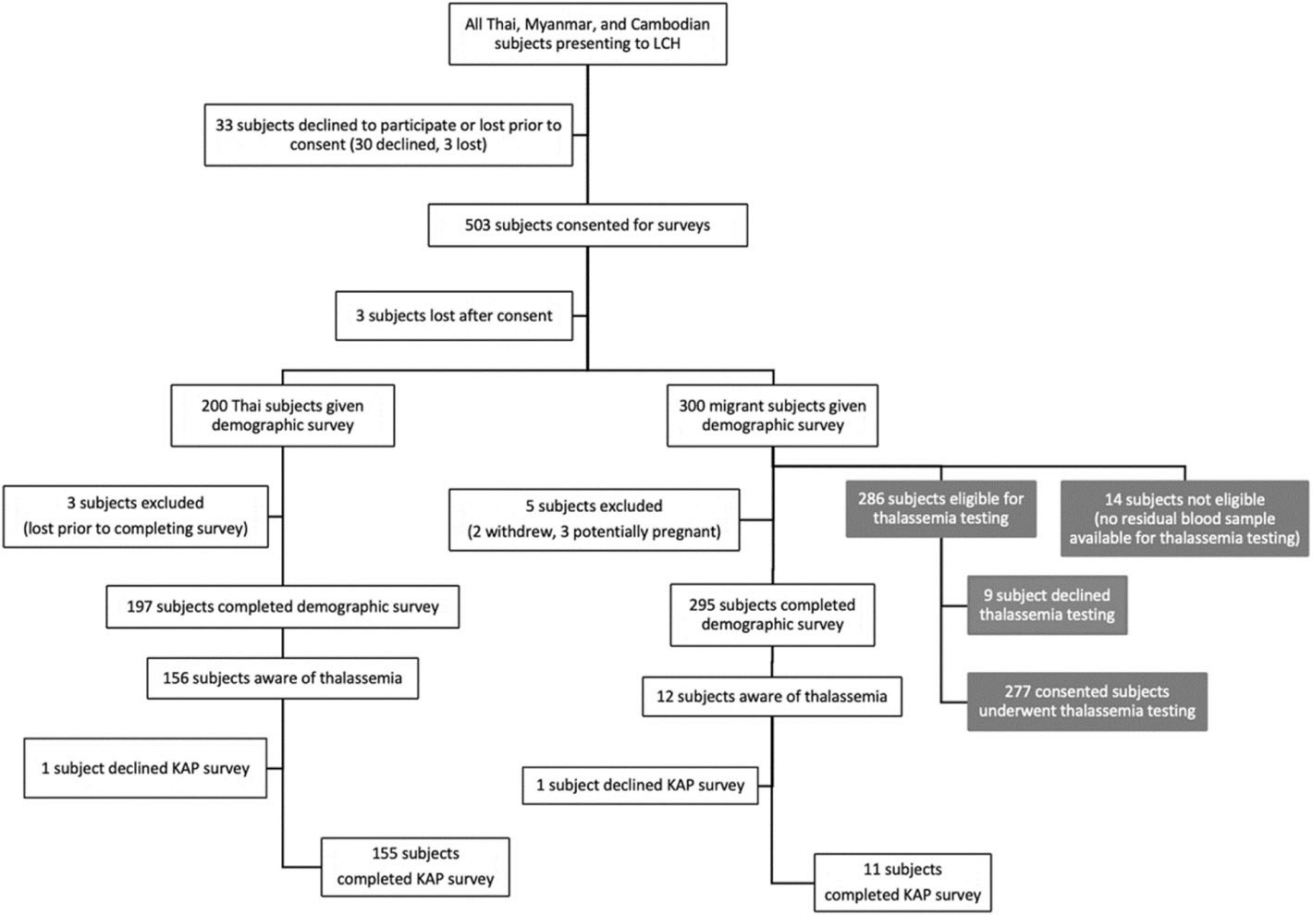
CONSORT flow diagram for surveys and thalassemia testing (the latter in gray). Thai, Myanmar, and Cambodian subjects of reproductive age (18–49 years old) presenting to Laem Chabang Hospital (LCH) were screened for eligibility. Those individuals who did not speak Thai, Burmese, or Khmer, or pregnant or with a pregnant partner by self-report, were excluded. Subjects were considered lost if they were approached by the study team and initially agreed to participate in the study, but subsequently left the hospital before completing the study without alerting the study team. Three subjects were lost prior to consent, 3 following consent and prior to starting the surveys, and 3 prior to completing the full demographic survey. Out of 200 Thai and 300 migrant subjects surveyed, 492 subjects completed the demographic survey. The KAP survey was completed by 155 Thai and 11 migrant subjects who were aware of thalassemia. Out of the 300 migrant subjects offered thalassemia testing, 286 were eligible for and 277 (96.9%) consented to thalassemia testing

We performed a cross-sectional study of Myanmar and Cambodian migrant and Thai subjects of reproductive age (18–49 years) residing in Chonburi, from February to May 2018 at Laem Chabang Hospital (LCH), a secondary care center serving migrants in its catchment area under the Ministry of Public Health. Migrants were screened for eligibility at the time of presentation for health registration (either renewal or new registration), an annual prerequisite for obtaining a work permit and legal status. Thai subjects presenting for non-urgent outpatient care at LCH were screened for eligibility and used as a comparison population for surveys. Thai, Burmese, and Khmer language interpreters were used, and only subjects who spoke one of these languages were eligible. Those pregnant or with a pregnant partner by self-report were excluded. A total of 200 Thai and 300 migrant subjects were surveyed. A small number of subjects were lost before or after providing consent (Fig. 1). A lost subject was defined as an individual who initially agreed to participate in the study, but subsequently left the hospital before completing the study without alerting the study team of their withdrawal from the study; these individuals were excluded from the analysis.

### Survey development and administration

Demographic and KAP surveys were developed using existing literature on thalassemia-related surveys [18–22] and expert opinion. Surveys were translated from English to Thai, Burmese, and Khmer and assessed for face and ethnographic validity by the research team and by Myanmar and Cambodian providers and interpreters (Appendix 1A-D in Additional file 1). “Nationality” was used in place of ethnicity due to the existence of ethnic minorities that complicate the definition of ethnicity. In assessing awareness of thalassemia, the medical term “thalassemia” was used, as no idiom or layman’s term exists in Thai, Burmese, or Khmer based on expert opinion.

Awareness of thalassemia was the primary outcome, with demographic characteristics, health-seeking behaviors, personal and family medical history, and awareness of anemia used as predictors. All subjects completed the 32-question demographic survey. Only subjects reporting awareness of thalassemia completed the 31-question KAP survey, consisting of three domains: 12 true-or-false knowledge questions, 14 attitude questions based on a 5-point Likert scale, and five nested questions about practices surrounding thalassemia testing and reproduction (Appendix 2A-D in Additional file 1). Surveys were administered to the majority of migrants orally with interpreter assistance, due to lower rates of literacy compared to Thai subjects.

### Sample size calculation and statistical methods

Based on published differences in awareness in migrants or ethnic minorities compared to natives [23], we hypothesized at least a 25% difference in thalassemia awareness between Thai and migrant subjects. Utilizing a sample size of 200 per group, we expected to achieve 95% power, assuming α = 0.05. We allowed for an increase in the enrollment target to 300 if < 30 subjects (15%) in either group completed the KAP survey.

Descriptive statistics were used to compare sociodemographic characteristics. Subgroup analyses for predictors of thalassemia awareness were performed in Thai and migrant subjects. Associations between awareness of thalassemia and predictors were assessed using Chi-Square analysis, Fisher’s Exact Test, and univariate logistic regression. Descriptive statistics were used to compare individual KAP survey questions. The knowledge score reflected the number of correctly answered knowledge questions out of 12. Attitude questions were scored using the Likert scale, with inverse questions scored in reverse, and averaged to obtain an attitude score. Medians with inter-quartile ranges (IQR) were reported for knowledge and attitude scores, and non-parametric tests were used to compare medians between Thai and migrant groups. The correlation between knowledge and attitude scores was assessed using the Spearman correlation for non-parametric data. Cronbach’s alpha was used to assess the internal reliability of both the knowledge and attitude sections. The level of statistical significance was set as *p* < 0.05. All statistical analyses were performed using SAS software, version 9.4 (SAS Institute Inc., Cary, NC, USA).

### Laboratory and molecular testing for thalassemia

Thalassemia testing was offered to migrants in a separate informed consent process after completion of surveys, so as not to bias subjects’ perspective on thalassemia screening or awareness/knowledge. For all migrant subjects consenting to thalassemia testing, complete blood count (CBC) was performed using an automated red blood cell counter (Sysmex F280, Sysmex, Tokyo, Japan), and Hb typing was performed by high-performance liquid chromatography (HPLC) using Variant II (Bio-Rad Laboratories, Hercules, CA, USA). Multiplex Gap-polymerase chain reaction (PCR) and amplification-refractory mutation system-PCR assays for the detection of common α-thalassemia mutations (listed in Table 2) was performed for all samples [24, 25], Hb E variants were identified by elevated Hb A_2_/E by HPLC in the expected percent ranges for heterozygosity or homozygosity Hb E (25–35% for Hb E trait without α-thalassemia; 15–20% for Hb E trait with α-thalassemia; > 80% for Hb E homozygote). All subjects with elevated Hb A_2_ ≥ 3.5% (but lower than the expected ranges for Hb E listed above) underwent multiplex PCR for 16 common β-globin gene mutations (listed in Table 2) as per previously published techniques [24]. The frequency of thalassemia and Hb variants in our migrant cohort was compared to a separate Thai community cohort that was genotyped using the same molecular approach. This larger Thai cohort (*N* = 1232) was composed of subjects aged 3–90 years (median age of 28 years; interquartile range of 20–40 years; 69.8% female) who resided in Chonburi.

**Table 2:** Common α- and β-globin mutations identified using previously described multiplex PCR-based techniques [24].

No.	Globin gene type	Mutation	Mutation type
1	α-globin	-α^3.7^	Single deletion
2		-α^4.2^	Single deletion
3		- -^SEA^	Double deletion
4		- -^THAI^	Double deletion
5		- -^FIL^	Double deletion
6		- -^MED^	Double deletion
7		-(α)^20.5^	Double deletion
8		- -^SIAM^	Double deletion^a^
9		Initiation codon (ATG > A-G)	Non-deletion
10		Codon 30 (∆GAG)	Non-deletion
11		Codon 59 (GGC > GAC)	Non-deletion
12		Codon 142 (Pakse; TAA > TAT)	Non-deletion
13		Codon 125 (Quang Sze; CTG > CCG)	Non-deletion
14		Codon 142 (Constant Spring; TAA > CAA)	Non-deletion
15	β-globin	nt −28 (A > G)	β^+^
16		Codon 8/9 (+G)	β^0^
17		IVSI-5 (G > C)	β^+^
18		Codon 41/42 (−TTCT)	β^0^
19		Codon 71/72 (+A)	β^0^
20		Codon 17 (A > T)	β^0^
21		IVSI-1 (G > T)	β^0^
22		Codon 26 Hb E (GAG > AAG)	β^E^
23		IVSII-654 (C > T)	β^+^
24		Codon 35 (C > A)	β^0^
25		Codon 43 (G > T)	β^0^
26		Codon 26 (G > T)	β^0^
27		Codon 95 (+A)	β^0^
28		Codon 19 (A > G)	β^+^
29		Codon 41 (−C)	β^0^
30		Codon 27/28 (+C)	β^0^

^a^ The α-globin deletion - -^SIAM^ was identified using the methodology described in Riolueang et al. 2019 [25].

### Projected rates of births affected by severe thalassemia

Birth rates, or the annual number of newborns affected by severe thalassemia per 1000 births, were calculated using the Hardy-Weinberg equation similar to previously described methods [26, 27]. Briefly, the allele frequencies of ⍺^0^-thalassemia (*p*; − -^SEA^ and - -^THAI^), deletional ⍺^+^-thalassemia (*q*; −⍺^3.7^and -⍺^4.2^), and non-deletional ⍺^+^-thalassemia (*r*; ⍺^CS^⍺, ⍺^PS^⍺, ⍺^QZ^⍺, and ⍺^WM^⍺) variants found in Thai, Myanmar, and Cambodian cohorts were used to calculate the expected frequencies of Hb Bart’s hydrops fetalis (*p*^2^), all Hb H disease (2*p(q + r)*), deletional Hb H disease (2*pq*), and non-deletional Hb H disease (2*pr*) for each nationality. For β-thalassemia, the frequencies of homozygous β-thalassemia ((*s + t)*^2^), all Hb E/β-thalassemia (2(*s* + *t*)*u*), Hb E/β^+^-thalassemia (2*su*), and Hb E/β^0^-thalassemia (2*tu*) were similarly calculated from the allele frequencies of β^+^-thalassemia (*s*), β^0^-thalassemia (*t*), and Hb E (*u*) variants. Hb D was excluded from this analysis. To calculate projected birth rates in each SEA population, the 2015–2020 crude birth rate from the 2019 United Nations World Population Prospects [28] and the numbers of migrant workers in Thailand from Thai Foreign Worker Administration Office [29] were used. Consanguinity was not considered in the calculations due to strong cultural norms discouraging consanguineous marriages among the Thai population [30].

## Results

There were 197 Thai, 119 Myanmar, and 176 Cambodian (492 total) subjects (Fig. 1 and Table 3). The median age was similar among all groups, but the Cambodian cohort had more males. Over 70% of migrants reported some proficiency in Thai. Around 60 and 27% of Thai space obtained secondary and higher education, respectively, compared to 35 and 3% of Myanmar and 30 and 1% of Cambodians. Cambodians had the highest proportion of subjects (~ 27%) with no formal education. Migrants were more often married than Thai subjects but reported similar numbers of children (Table 3). Marriage to an individual with a different nationality was reported by ~ 5% of migrants (with 8/11 having a Thai spouse), while Thai subjects reported no inter-group marriages.

**Table 3:** Characteristics of the study population

	Thai	Myanmar	Cambodian
	(***N*** = 197)	(***N*** = 119)	(***N*** = 176)
Median Age (IQR), years	32 (24–40)	29 (24–36)	30 (27–37)
Gender			
Male (%)	71 (36.0)	43 (36.4)	100 (56.8)
Female (%)	126 (64.0)	75 (63.6)	76 (43.2)
Languages Spoken^a^			
Thai (%)	196 (99.5)	73 (61.3)	133 (76.0)
Burmese (%)	0	109 (91.6)	7 (4.1)
Khmer (%)	0	2 (1.7)	168 (97.1)
English (%)	49 (24.9)	0	3 (1.7)
Mon (%)	1 (0.5)	7 (5.9)	0
Karen (%)	0	9 (7.6)	0
Highest Level of Education Achieved			
None (%)	2 (1.0)	18 (15.4)	48 (27.4)
Primary, 1–6 (%)	22 (11.3)	53 (45.3)	74 (42.3)
Secondary, 7–9 (%)	58 (29.9)	29 (24.8)	41 (23.4)
Secondary 10–12 (%)	58 (29.9)	12 (10.3)	11 (6.3)
Vocational (%)	24 (12.4)	0	0
Bachelor’s degree and above (%)	28 (14.4)	3 (2.6)	1 (0.6)
Work Sector			
Fishery (%)	2 (1.1)	0	2 (1.2)
Manufacturing (%)	63 (34.6)	40 (36.0)	75 (43.1)
Domestic (%)	40 (22.0)	37 (33.3)	13 (7.5)
Construction (%)	5 (2.8)	8 (7.2)	43 (24.7)
Other (%)^b^	72 (39.6)	26 (23.4)	41 (23.6)
Median Duration in Thailand (IQR), years	NA	6 (4.25–10)	5 (3–6)
Median Number of Times Moved (IQR)	NA	0 (0–2)	0 (0–2)
Health Registration			
First time (%)	NA	28 (25.0)	42 (24.1)
Renewal (%)	NA	84 (75.0)	132 (75.9)
Marital Status			
Single (%)	65 (33.9)	32 (27.1)	39 (22.7)
Married (%)	114 (59.4)	83 (70.3)	127 (73.8)
Divorced (%)	13 (6.8)	3 (2.5)	6 (3.5)
Nationality of Spouse if Married			
Thai (%)	111 (100)	3 (3.6)	5 (4.0)
Myanmar (%)	0	75 (93.8)	2 (1.7)
Cambodian (%)	0	1 (1.3)	113 (94.2)
Median Number of Children (IQR)	2 (1–2)	1 (0–2)	2 (1–2)

*IQR* Inter-quartile range, *NA* Not applicable

^a^ Ethnic minority languages were reported by the Myanmar cohort, including Mon (5.9%), Karen (7.6%), and Chinese (0.8%), but not by the Thai or Cambodian cohorts

^b^ The large majority of jobs in the “Other” category were reported to be sales-related, with job descriptions ranging from company salesmen to street vendors. Other job sectors included business, civil, and farming. Some subjects reported being students or freelance/self-employed

Myanmar subjects reported residing in Thailand for longer than Cambodians. Nevertheless, transience was surprisingly low, with the majority having never moved within Thailand. Within work sectors (Table 3), more Thai subjects reported holding white-collar jobs (e.g., managerial or sales), while migrants largely held blue-collar or informal sector jobs. Three-quarters of migrant workers had previously undergone health registration, while one-quarter reported registering for the first time (i.e., were likely undocumented or “unregistered”).

### Healthcare utilization is lower among migrants

Most subjects preferred to first seek care from a doctor or medical facility. Drug stores were the next most preferred contact, while few reported seeking care from traditional healers (Table 4). Thai and Cambodian subjects relied heavily on public health insurance. Migrants generally used cash more often than Thai subjects to pay for health care (~ 36% vs. 11%). Thai subjects had a higher median number of doctor visits within the past 1 year compared to migrants (Table 4). Unregistered status was associated with shorter length of residence (median 4.0 years, vs. 5.1 years for registered migrants).

**Table 4:** Comparison of thalassemia-related health literacy and health behaviors among Thai, Myanmar, and Cambodian subjects

	Thai	Myanmar	Cambodian
	(***N*** = 197)	(***N*** = 119)	(***N*** = 176)
**Health-seeking Behaviors**			
First Point of Contact When Sick			
Drug store (%)	71 (37.6)	47 (40.2)	77 (44.8)
Doctor (%)	115 (60.9)	65 (55.6)	88 (51.2)
Traditional healer (%)	1 (0.5)	3 (2.6)	3 (1.7)
Other (%)^a^	2 (1.1)	2 (1.7)	4 (2.3)
Median Number of Doctor Visits in 1 year (IQR)	1 (0–3)	0 (0–1)	0 (0–2)
Method of Payment for Healthcare			
Public Insurance (%)	82 (44.1)	37 (31.4)	80 (46.2)
Employer Insurance (%)	52 (28.0)	29 (24.6)	30 (17.3)
Private Insurance (%)	18 (9.7)	1 (0.9)	1 (0.6)
Cash (%)	19 (10.2)	49 (41.5)	53 (30.6)
Other (%)^b^	15 (8.1)	2 (1.7)	9 (5.2)
Want to test self for thalassemia (%)^c^	25 (62.5)	67 (61.5)	147 (86.5)
Want to test baby for thalassemia (%)^c^	29 (72.5)	77 (70.6)	145 (86.8)
Want to test partner for thalassemia (%)^c^	29 (78.4)	81 (74.3)	150 (88.8)
Best Method for Thalassemia Education			
Consultation with doctor (%)	26 (68.4)	93 (86.9)	144 (84.2)
Brochure (%)	1 (2.6)	8 (7.5)	10 (5.9)
Employer workshop (%)	1 (2.6)	1 (0.9)	2 (1.2)
Government workshop (%)	5 (13.2)	2 (1.9)	5 (2.9)
Other (%)^d^	5 (13.2)	3 (2.8)	10 (5.9)
**Thalassemia-related Health Literacy**			
Aware of Anemia (%)	151 (80.3)	23 (20.0)	24 (13.7)
Aware of Thalassemia (%)	156 (79.6)	8 (6.8)	4 (2.3)
Personal History of:			
Anemia (%)	31 (16.0)	7 (6.0)	7 (4.1)
Thalassemia (%)	12 (6.2)	0	3 (1.7)
Transfusion (%)	13 (6.7)	1 (0.9)	10 (5.8)
Miscarriage (%)	15 (7.9)	8 (6.9)	11 (6.4)
Family History of:			
Anemia (%)	34 (17.4)	4 (3.5)	9 (5.2)
Thalassemia (%)	11 (5.7)	1 (0.9)	1 (0.6)
Transfusion (%)	15 (7.8)	2 (1.7)	6 (3.5)
Miscarriage (%)	17 (8.9)	5 (4.4)	8 (4.6)

*IQR* Inter-quartile range

^a^ Other preferred sources of medical care included on-site clinics provided by the employer in workplaces such as factories, or multiple sources of medical care were selected

^b^ Other methods of payment include civil insurance and the selection of multiple sources of medical care

^c^ Subjects unaware of thalassemia were asked whether they would want to test themselves, their babies, or their partners for an inherited disease, such as thalassemia

^d^ Other preferred methods of education included mass media (television, internet), word of mouth (friends, employers, spouse), or multiple sources

### Migrants have very low thalassemia awareness but show interest in thalassemia screening

Most Thai subjects were aware of anemia and thalassemia (both ~ 80%). In contrast, a minority of Myanmar and Cambodian migrants were aware of anemia (20 and 13.7%), and few were aware of thalassemia (6.8 and 2.3%). Thai subjects also reported more personal and family history of anemia, thalassemia, and blood transfusion compared to migrants (Table 4).

Subjects without awareness of thalassemia were briefly informed about thalassemia and asked if they were interested in thalassemia screening. Most expressed interest, with Cambodians showing greatest interest. All groups preferred to receive thalassemia education through consultations with a doctor, with migrants showing a stronger preference (Table 4).

### Factors associated with thalassemia awareness

Subgroup analysis of factors predicting thalassemia awareness in migrants revealed an association with longer residence duration in Thailand (median 13.1 vs. 5.0 years; Z = 3.82, *p* = 0.0002). Within the Thai subgroup, females had 2.12 times higher odds (95% CI 1.05–4.29; *p* = 0.03) of being aware of thalassemia compared to males, and those with secondary education had 2.69 times higher odds (95% CI 1.08–6.71, p = 0.03) than those less educated. Among both Thai and migrant subjects, awareness of anemia strongly predicted thalassemia awareness (OR = 11.07 [95% CI 4.82–25.44, *p* < 0.0001] and 18.87 [95% CI 4.89, 72.84, p < 0.0001], respectively).

### Comparison of knowledge, attitudes, and practices of migrant vs. Thai subjects

Thalassemia knowledge was lower for migrants compared to Thai subjects (median knowledge score of 7 (IQR 6–8) vs. 8 (IQR 7–10), Z = -3.10, *p* = 0.002, Table 5). Only 27.3% of migrants knew that thalassemia was common in Asia, vs. 72.3% of Thai subjects. Compared to 54.2% of Thai subjects, 90.9% of migrants thought a thalassemia carrier could develop thalassemia major. However, both groups held key misconceptions: 32.9% of Thai and 45.4% of migrant subjects believed that thalassemia was an infectious disease, and 39.3 and 54.5%, respectively, believed that thalassemia was curable with a pill rather than a chronic disease. Understanding of carrier status was poor; most Thai and migrant subjects believed that non-carrier couples could give birth to a child with thalassemia and that thalassemia carriers require blood transfusions. Subjects without secondary education had lower knowledge levels than those with secondary education (median score of 6 (IQR 6–9) vs. 8 (IQR 8–10), Z = 3.30, *p* = 0.001). Age, gender, and health-seeking behaviors did not correlate with knowledge scores.

**Table 5:** Individual question responses and cumulative knowledge and attitude scores from the KAP survey

	Thai	Migrant
	(***N*** = 155)	(***N*** = 11)
**Knowledge Questions (% Correct)**		
1. Thalassemia is blood disease.	94.8%	72.7%
2. Thalassemia is cause of anemia.	87.7%	81.8%
3. Thalassemia is rare in Asia.	72.3%	27.3%
4. Thalassemia is infectious disease.	67.1%	54.6%
5. Thalassemia is inherited disease.	91.0%	72.7%
6. Normal parents can have affected child.	25.8%	9.1%
7. Carrier parents at risk for affected pregnancy.	70.3%	63.6%
8. Blood test can be done.	97.4%	100.0%
9. Thalassemia carrier requires transfusions.	42.6%	36.4%
10. Thalassemia major requires transfusions.	86.5%	90.9%
11. A carrier can develop thalassemia major.	45.8%	9.1%
12. Can cure thalassemia with pill.	60.7%	45.5%
*Median Knowledge Score (IQR)* ^a^	*8 (7–10)*	*7 (6–8)*
**Attitude Questions (Median, IQR)**		
13. Thalassemia should be prevented.	4 (4–5)	5 (5–5)
14. Child with thalassemia is burden.	3 (2–4)	4 (2–5)
15. Child with thalassemia is blessing.	1 (1–2)	1 (1–2)
16. Want to know if having baby affected.	4 (4–5)	5 (4–5)
17. Would get tested during pregnancy.	5 (4–5)	5 (5–5)
18. Want partner tested during pregnancy.	5 (4–5)	5 (3–5)
19. Have children with partner if both carriers.	3 (2–4)	1 (1–3)
20. Would not want to know if baby affected.	2 (1–4)	1 (1–1)
21. Testing during pregnancy not useful.	3 (1–4)	1 (1–5)
22. Not have children with partner if both carriers.	3 (2–5)	1 (1–5)
23. Would end pregnancy for thalassemia.	3 (2–4)	2 (1–5)
24. Disagree with ending pregnancy.	4 (3–4)	5 (4–5)
25. Would not prevent birth of affected baby.	3 (3–4)	5 (1–5)
26. Better to end pregnancy than let child suffer.	4 (3–4)	3 (1–5)
*Median Attitude Score (IQR)* ^b^	*3.4 (3.2–3.8)*	*3.7 (3.3–3.9)*
**Practice Questions (N, %)**		
27. Subject tested for thalassemia.	72 (46.8)	3 (27.3)
28. Partner tested for thalassemia.	59 (38.3)	1 (9.1)
29. Had prenatal diagnosis (PND).	53 (68.0)	2 (66.7)
30. PND showed affected baby.	4 (7.6)	1 (50.0)
31. Plan to have more children.	94 (62.7)	8 (72.7)

^a^ The Knowledge Score was defined as the number of correctly answered knowledge questions (out of 12 possible points). The median Knowledge Score was higher in Thai vs migrant subjects (p = 0.002)

^b^ The Attitude Score was defined as the mean value of 14 attitude questions scored according to the Likert scale (Strongly agree = 5 points, Agree = 4, Unsure = 3, Disagree = 2, and Strongly disagree = 1), with inverse questions scored in reverse. There was no statistical difference between median Attitude Scores among Thai vs migrant subjects (*p* = 0.21)

Attitude scores were considered a proxy for subjects’ attitudes towards thalassemia prevention and control, with a score of 5 indicating strong support and 1 indicating strong opposition. The median attitude score was 3.4 (IQR 3.2–3.8) for Thai subjects and 3.7 (IQR 3.3–3.9) for migrants, suggesting that subjects leaned towards supporting thalassemia prevention overall. Examining individual questions, Thai and migrant subjects agreed that thalassemia should be prevented, that having a child with thalassemia was more of a burden than a blessing, and that they preferred to know whether their pregnancy was affected by thalassemia and would test themselves and their partners (Table 5). Paradoxically, migrants expressed a stronger desire to know whether a pregnancy was affected by thalassemia (Questions 16 and 20) but held more negative attitudes toward actual termination of pregnancy (Questions 23–26, Table 5). No correlation between knowledge and attitude scores was found. Cronbach’s ⍺ for internal reliability of the survey tool was 0.66 for knowledge questions and 0.67 for attitude questions.

Finally, Thai subjects and their partners reported having been tested for thalassemia more often than migrants (Table 5). Of those tested, around two-thirds reported undergoing prenatal diagnosis (PND), leading to a diagnosis of severe thalassemia in one migrant pregnancy and four Thai pregnancies; the decision for termination of pregnancy was not captured. Most subjects in both groups planned to have more children in the future (Table 5).

### Thalassemia is prevalent in migrants, and screening is feasible and acceptable

Of the 300 migrants surveyed, 286 subjects (95.3%) underwent phlebotomy and had residual blood samples available for thalassemia testing, demonstrating the feasibility of obtaining blood samples for thalassemia testing during the health registration process. Of the 286 subjects, 277 (96.9%) consented to thalassemia testing, suggesting that thalassemia testing is acceptable to migrants. We identified at least one ⍺- or β-globin mutation in 53.4% (148/277) of migrants, compared to 43.8% (540/1232) of Thai subjects in a separate cohort (Table 6).

**Table 6:** Allele frequencies of the most common hemoglobin variants identified in Thai vs. migrant cohorts

		Migrant		
	Thai	Myanmar	Cambodian	Combined
	(***N*** = 1232)	(***N*** = 121)	(***N*** = 156)	(***N*** = 277)
**Deletional ⍺**^**+**^**-thalassemia**	**0.088**	**0.186**	**0.183**	**0.184**
⍺^3.7^	0.080	0.182	0.179	0.181
-⍺^4.2^	0.008	0.004	0.003	0.004
**Deletional ⍺**^**0**^**- thalassemia**	**0.022**	**0.004**	**0.013**	**0.009**
- -^SEA^	0.022	0.004	0.010	0.007
- -^THAI^	0	0	0.003	0.002
**Non-deletional ⍺**^**+**^**- thalassemia**	**0.026**	**0**	**0.003**	**0.002**
Hb Constant Spring	0.024	0	0	0
Hb Pakse	0.001	0	0.003	0.002
**Hb E**	**0.127**	**0.058**	**0.192**	**0.134**
**β-thalassemia (β**^**0**^ **or β**^**+**^**)**	**0.007**	**0.025**	**0.003**	**0.013**
β^IVSI-1^	0.001	0.012	0.003	0.007
β^CD17^	0.002	0	0	0
β^CD71/72^	0.0004	0.004	0	0.002
β^CD41/42^	0.001	0	0	0

The most common variants identified were -⍺^3.7^ and Hb E. The Myanmar and Cambodian cohorts had a higher frequency of deletional ⍺^+^-thalassemia alleles (0.186 and 0.183, respectively) compared to the Thai cohort (0.088). Hb E allele frequency was highest amongst Cambodians (0.192), and β-thalassemia allele frequency highest amongst Myanmar (0.025). Non-deletional ⍺-globin variants (Hb Constant Spring, Hb Pakse, and Hb Quang Sze) and deletional ⍺^0^-thalassemia variants (− −^SEA^ and - -^THAI^) were more frequent in the Thai cohort. Around 15% of Cambodians were double heterozygous carriers of ⍺- and β-globin variants due to high frequencies of Hb E and -⍺^3.7^, compared to 4–5% of the Thai and Myanmar cohorts (Table 7). Severe thalassemia disease syndromes (11, Hb H disease; 1, Hb E/β-thalassemia) were only identified in the Thai cohort. Rare ⍺- and β-globin variants were found in Thai (Hb Westmead, Hb Quang Sze, and Hb D) and migrant (Hb Showa-Yakushiji) cohorts (Table 7).

**Table 7:** ⍺- and β-globin genotypes identified in Thai and migrant cohorts in Chonburi, Thailand

Disease Category	Genotype			Migrants					
		Thai (***N*** = 1232)		Myanmar (***N*** = 121)		Cambodian (***N*** = 156)		Combined (***N*** = 277)	
		N	%	N	%	N	%	N	%
Carriers of ⍺-globin variant	-⍺^3.7^/⍺⍺	126	10.2	34	28.1	30	19.2	64	23.1
	-⍺^4.2^/⍺⍺	13	1.1	1	0.8	0	0	1	0.4
	-⍺^3.7^/−⍺^3.7^	4	0.3	2	1.7	1	0.6	3	1.1
	-⍺^3.7^/−⍺^4.2^	1	0.1	0	0	1	0.6	1	0.4
	-⍺^3.7^/⍺^CS^⍺	8	0.6	0	0	0	0	0	0
	- -^SEA^/⍺⍺	34	2.8	1	0.8	2	1.3	3	1.1
	⍺^CS^⍺/⍺⍺	35	2.8	0	0	0	0	0	0
	⍺^PS^⍺/⍺⍺	1	0.1	0	0	0	0	0	0
	⍺^QZ^⍺/⍺⍺	1	0.1	0	0	0	0	0	0
	**Total**	**223**	**18.1**	**38**	**31.4**	**34**	**21.8**	**72**	**26.0**
Carriers of β-globin variant	β^E^/β	186	15.1	10	8.3	29	18.6	39	14.1
	β^D^/β	3	0.2	0	0	0	0	0	0
	β^IVSI-1^/β	1	0.1	1	0.8	1	0.6	2	0.7
	β^IVSII-654^/β	1	0.1	0	0	0	0	0	0
	β^CD17^/β	5	0.4	0	0	0	0	0	0
	β^CD71/72^/β	1	0.1	1	0.8	0	0	1	0.4
	β^CD41/42^/β	1	0.1	0	0	0	0	0	0
	β^CD27/28^/β	1	0.1	0	0	0	0	0	0
	β^−28^/β	1	0.1	0	0	0	0	0	0
	β^-3.48^/β	2	0.2	1	0.8	0	0	1	0.4
	**Total**	**202**	**16.4**	**13**	**10.7**	**30**	**19.2**	**43**	**15.5**
Double heterozygous carriers	-⍺^3.7^/⍺⍺ + β^E^/β	28	2.3	2	1.7	18	11.5	20	7.2
	-⍺^4.2^/⍺⍺ + β^E^/β	6	0.5	0	0	0	0	0	0
	-⍺^3.7^/−⍺^3.7^ + β^E^/β	5	0.4	0	0	2	1.3	2	0.7
	- -^SEA^/⍺⍺ + β^E^/β	8	0.6	0	0	1	0.6	1	0.4
	- -^THAI^/⍺⍺ + β^E^/β	0	0	0	0	1	0.6	1	0.4
	⍺^CS^⍺/⍺⍺ + β^E^/β	5	0.4	0	0	0	0	0	0
	⍺^PS^⍺/⍺⍺ + β^E^/β	0	0	0	0	1	0.6	1	0.4
	-⍺^3.7^/⍺⍺ + β^D^/β	1	0.1	0	0	0	0	0	0
	-⍺^3.7^/⍺⍺ + β^CD41/42^/β	1	0.1	0	0	0	0	0	0
	-⍺^3.7^/⍺⍺ + β^CD35^/β	1	0.1	0	0	0	0	0	0
	-⍺^3.7^/⍺⍺ + β^−28^/β	1	0.1	0	0	0	0	0	0
	-⍺^3.7^/⍺⍺ + β^IVSI-1^/β	0	0	2	0.8	0	0	2	0.7
	-⍺^3.7^/⍺⍺ + β^CD110^/β ^a^	0	0	1	0.8	0	0	1	0.4
	⍺^CS^⍺/⍺⍺ + β^IVSI-1^/β	1	0.1	0	0	0	0	0	0
	**Total**	**57**	**4.6**	**5**	**4.1**	**23**	**14.7**	**28**	**10.1**
Homozygous Hb E (with/without ⍺-thalassemia)	β^E^/β^E^	25	2.0	0	0	3	0.2	3	1.1
	-⍺^3.7^/⍺⍺ + β^E^/β^E^	4	0.3	1	0.1	1	0.1	2	0.7
	-⍺^3.7^/−⍺^3.7^ + β^E^/β^E^	1	0.1	0	0	0	0	0	0
	⍺^CS^⍺/⍺⍺ + β^E^/β^E^	3	0.2	0	0	0	0	0	0
	**Total**	**33**	**2.7**	**1**	**0.8**	**4**	**2.6**	**5**	**1.8**
Hb H disease (with/without ⍺-thalassemia)	- -^SEA^/−⍺^3.7^	6	0.5	0	0	0	0	0	0
	- -^SEA^/⍺^CS^⍺	3	0.2	0	0	0	0	0	0
	- -^SEA^/⍺^CS^⍺ + β^E^/β	1	0.1	0	0	0	0	0	0
	- -^SEA^/⍺^WM^⍺ + β^E^/β	1	0.1	0	0	0	0	0	0
	**Total**	**11**	**0.9**	**0**	**0**	**0**	**0**	**0**	**0**
Compound heterozygous β-thalassemia	β^CD41/42^/β^E^	1	0.1	0	0	0	0	0	0
	**Total**	**1**	**0.1**	**0**	**0**	**0**	**0**	**0**	**0**
Other ⍺-thalassemia diseases	⍺^CS^⍺/⍺^CS^⍺	1	0.1	0	0	0	0	0	0
	⍺^CS^⍺/⍺^PS^⍺ + β^E^/β	1	0.1	0	0	0	0	0	0
	⍺ triplication	5	0.4	NT	NT	NT	NT	NT	NT
	⍺ triplication + β^E^/β	6	0.5	NT	NT	NT	NT	NT	NT
	**Total**	**13**	**1.1**	**0**	**0**	**0**	**0**	**0**	**0**
**Any thalassemia carrier**	**Total**	**540**	**43.8**	**57**	**47.1**	**91**	**58.3**	**148**	**53.4**

*NT* Not tested, *CS* Constant Spring, *PS* Pakse, *QZ* Quang Sze, *WM* Westmead

^a^ β^CD110^ is also known as Hb Showa-Yakushiji

### Higher rate of births with severe thalassemia among migrant populations

Based on prevalence estimates from this study, the projected rate of births affected by all thalassemia diseases is 11.193/1000 births/year in migrants and 9.167/1000 births/year in the Thai population. Projected birth rates are highest for Hb Bart’s hydrops fetalis and Hb E/β-thalassemia in the Thai population and for homozygous β-thalassemia in the Myanmar migrant population (Table 8). Projected Hb H disease birth rates are similar in Thai and Cambodian migrant populations but lower in Myanmar migrants (4.885 and 4.759 vs. 1.525/1000 births/year, respectively). The projected number of newborns in the Thai population with Hb Bart’s hydrops fetalis and Hb H disease, adjusted for annual birth rate, is comparable to previous estimates [27], but the number of Thai newborns affected by β-thalassemia syndromes is higher than prior estimates [31] (Table 8).

**Table 8:** Comparison of estimates of affected newborns with thalassemia disease for Thai populations and migrant workers

	Thai (Current study)	Thai (Hockham et al., 2019) [27]	Thai (Fucharoen et al., 2014) [31]	Myanmar (Current study)	Cambodian (Current study)
Hb Bart’s hydrops fetalis					
• Affected newborns (cases/1000 birth/year)	0.462	N/A	N/A	0.017	0.164
• Affected newborns in Thailand^a^ (cases/year)	335	423 (CI: 184–761)	833	< 1 (0.18)	< 1 (0.23)
Hb H disease (all)					
• Affected newborns (cases/1000 birth/year)	4.885	N/A	N/A	1.525	4.759
• Affected newborns in Thailand^a^ (cases/year)	3540	3172	5600	16	7
• Deletional Hb H disease (cases/year)	2742	2674 (CI:1296–4491)	N/A	16	7
• Non-deletional Hb H disease (cases/year)	798	498 (CI:237–947)	N/A	0	0 (0.1)
Homozygous β-thalassemia					
• Affected newborns (cases/1000 birth/year)	0.202	N/A	N/A	0.615	0.010
• Affected newborns in Thailand^a^ (cases/year)	146	N/A	207	7	< 1 (0.01)
Hb E/β-thalassemia					
• Affected newborns (cases/1000 birth/year)	3.618	N/A	N/A	2.872	1.231
• Affected newborns in Thailand^a^ (cases/year)	2622	N/A	3213	31	2
• Hb E/β^+^-thalassemia (cases/year)	1126	N/A	N/A	5	0
• Hb E/β^0^-thalassemia (cases/year)	1496	N/A	N/A	26	2

*NA* Not available, *CI* 95% confidence interval

^a^ Estimated numbers of births in Thailand in 2020 are 10,765 and 1418 for Myanmar and Cambodian migrant workers, respectively, and 724,587 for Thai population [28]

## Discussion

This is the first study characterizing thalassemia knowledge, attitudes, and health-related behaviors of migrant populations in Thailand and one of few studies examining beliefs and perceptions surrounding thalassemia in SEA. Through comprehensive thalassemia testing, we identified a higher proportion of thalassemia carriers and higher allele frequencies of some globin variants in a migrant cohort compared to a Thai cohort. Furthermore, using prevalence estimates, we found a higher projected rate of births affected by severe thalassemia in the migrant cohort compared to the Thai cohort, highlighting the significant disease burden in migrants that needs to be addressed.

We hypothesized existence of a significant gap in thalassemia awareness between Thai and migrant subjects; indeed, only 4% of migrants vs. 80% of Thai subjects had heard of thalassemia. Thalassemia awareness was associated with female gender and achieving secondary education in the Thai cohort. Our migrant cohort, on average, was more male and less educated. Consistent with migrants’ length of residence in Thailand being a proxy for linguistic integration and exposure to mass media, migrants aware of thalassemia had resided in Thailand longer. Additional potential factors leading to increased awareness in long-term migrant residents include increased likelihood of interaction with the healthcare system and larger social networks, in which knowledge of thalassemia may spread by word of mouth.

A study of thalassemia awareness among Italians vs. Italian-Americans also found a large knowledge gap (85% vs. 19%), attributed to ﻿exposure to thalassemia education and screening in Italy and the lack of such programs in the United States [23]. A similar disparity in exposure may underlie our findings, as Thailand’s thalassemia prevention and control programs only offer education, outreach, and free prenatal screening to Thai citizens and are not accessible to migrants. Education is an effective approach to lowering the burden of thalassemia [23, 32], and as such, bolstering education efforts in migrant communities could be a rational initial approach to addressing this awareness gap. However, educational efforts may be hindered by sociocultural barriers.

Education level and literacy were lower for migrant than Thai subjects in this study. Awareness of thalassemia was correlated with attainment of secondary education, as well as with awareness of hematological disorders in general, a measure of health literacy. Migrants also demonstrated a lower understanding of the genetic inheritance of thalassemia. A study in Yangon, Myanmar, found that even among mothers of children receiving regular care for thalassemia, only 18–28% had an understanding of genetic inheritance [19]. Therefore, effective thalassemia programs for migrant populations should also emphasize genetic education and counseling. In addition, Thai females were more aware of thalassemia than males, possibly related to Thailand’s prenatal approach to thalassemia prevention, providing women with more exposure through antenatal care clinics. For male migrant workers, alternative settings for screening and education should be explored.

Our study also highlights the significant structural barriers faced by migrant populations. One-quarter of our migrant cohort was unregistered, i.e., without legal documentation or access to public health insurance, potentially influencing their health seeking behaviors. Migrants were more likely to pay for healthcare with cash and less likely to have multiple types of insurance compared to Thai subjects, likely leading to higher out-of-pocket costs and lower healthcare utilization. Cost of thalassemia testing was a commonly raised concern among migrants, underscoring the need for a government-sponsored program for thalassemia prevention for migrants, similar to that for Thai citizens.

Other structural barriers related to employment, transportation, and lack of interpreters for migrants were frequently encountered during this study. Subjects undergoing thalassemia testing were offered a return of results through either domestic mail or in-person pick-up at LCH, as well as free thalassemia genetic counseling at LCH. The vast majority of migrants expressed interest in learning their thalassemia carrier status and requested the return of results but declined to return for counseling due to concerns over missing work or finding transportation. Despite having translated study documents to Thai, Burmese, and Khmer, we faced tremendous language barriers due to poor literacy among migrants, as well as the limited availability and quality of interpreters, which likely impacted subjects’ comprehension of more complex or inverted survey items (e.g., Questions 19 and 22, Table 5). Addressing structural barriers hindering the delivery of thalassemia prevention and care to migrants may require concurrent changes in immigration and employment policies, particularly as half of the migrant population in Thailand may be unregistered [33].

Additionally, the KAP survey revealed important misconceptions and beliefs that may reduce the acceptance of thalassemia screening. A large percentage of migrants believed thalassemia to be an infectious disease; correction of this and other misconceptions is required to prevent stigmatization of patients and carriers. Most migrants failed to identify thalassemia as a serious chronic condition and a common disease in Asia, which may detract from the perceived importance and urgency of screening. The fact that many Thai subjects held these same misconceptions highlights existing gaps in Thailand’s thalassemia education program that should be addressed.

All nationalities, in particular Cambodians, expressed a strong interest in the concept of thalassemia screening. However, migrants expressed more negative attitudes towards pregnancy termination, which might preclude the prenatal approach to thalassemia prevention in migrants. The surveys were not designed to elucidate reasons underlying migrants’ attitudes towards pregnancy termination; these themes should be explored in greater depth using qualitative methods, such as in-depth interviews and focus group discussions. On the other hand, our study had extremely high uptake (96.9%) of thalassemia screening by healthy migrant subjects, strongly suggesting that carrier screening would be acceptable to migrants.

Our health registration-based approach to thalassemia screening represents a unique and particularly efficient means of recruiting both documented and undocumented migrants. We found that public hospitals were a feasible, convenient setting for performing thalassemia screening but not for the return of results. An alternative setting that overcomes some structural barriers is the workplace, though privacy concerns and the willingness of employers to participate would need to be considered. Our study design has some limitations; survey results and thalassemia allele frequencies obtained from migrant workers presenting to LCH may not be representative of other migrant populations in Thailand. Indeed, we previously demonstrated spatial specificity in the gene frequency of ⍺-thalassemia in Thailand [27]. A similar geographic limitation may exist for the Thai community cohort undergoing thalassemia testing. The Thai cohort presenting to LCH is likely representative of the majority of the Thai population, which utilizes public health services due to the availability of universal health coverage in Thailand. However, individuals using the private health sector may differ in socioeconomic status and may not be represented in our cohorts. Additionally, due to the extremely low level of thalassemia awareness among migrants, only 11 migrant subjects completed the KAP survey, compared to 155 Thai subjects. The limited KAP survey data from migrants made drawing comparisons challenging and further subset analyses impossible. Ideally, our findings would be confirmed using a larger representative sample of migrants, employing either a multicenter approach or with comparisons to SEA migrant populations residing in other host countries.

Nevertheless, this study highlights the dramatic gap in thalassemia awareness between Thai citizens and migrants and the urgent need for public educational interventions accessible to migrants. Furthermore, we developed a multilingual KAP survey with face validity and acceptable reliability for assessing thalassemia knowledge and attitudes across three different nationalities (see Additional file 1). This thalassemia-specific KAP survey should be further validated in future studies for use as a multinational tool in SEA populations worldwide.

Finally, our data confirm the high prevalence of thalassemia among migrants in Thailand, laying the foundation for additional epidemiological and cost-effectiveness studies needed to inform future policy decisions surrounding thalassemia prevention and control. The projected annual number of migrant newborns affected by severe thalassemia is likely an underestimate, due to unregistered migrant populations being unaccounted for in official migration statistics, demonstrating the need for regularization of the migrant workforce to create effective public health policies.

## Conclusions

In this study, we developed a knowledge, attitudes, and practices (KAP) survey focused on thalassemia screening and validated the survey in healthy Myanmar, Cambodian, and Thai populations. We also offered comprehensive thalassemia testing to all migrant subjects and found that thalassemia is prevalent among Myanmar and Cambodian migrants and that nearly all migrants were willing to undergo thalassemia screening. However, we identified an extreme lack of awareness and understanding of thalassemia in migrant compared to Thai populations, as well as identified key misconceptions in both migrant and Thai populations that need to be addressed. Based on the KAP survey results, we conclude that lack of awareness of thalassemia is a prominent obstacle to thalassemia screening in migrant populations in Thailand and that public education efforts tailored to migrant populations are desperately needed. The negative attitudes towards termination of pregnancy among migrants suggest that Thailand’s prenatal approach to thalassemia screening may not be optimal for migrant populations and that other potential screening approaches should be explored. Indeed, we found carrier screening among migrant workers to be highly feasible and acceptable. Furthermore, the high frequencies of thalassemia variants identified in our migrant cohort argue strongly for the inclusion of migrant populations in thalassemia prevention and control policies in Thailand as well as regionally.

With increasing economic integration and cooperation among the ASEAN (Association of Southeast Asian Nations) member countries, intra-regional migration will continue to grow. The scientific community, healthcare providers, and policymakers must recognize the challenges posed by migration to regional thalassemia prevention and control and respond accordingly. Concerted efforts should be made not only to establish regional thalassemia screening programs and registries but also to extend thalassemia services to migrants within the region. With continued investigation into knowledge, cultural, and structural barriers to thalassemia prevention in migrant populations, we may begin to create a global solution for the growing public health problem of thalassemia.

## Supplementary Information

**Additional file 1.** Survey tools for thalassemia, Demographic and KAP surveys developed for this study are provided in English, Thai, Burmese, and Khmer languages.

## Data Availability

The datasets generated and/or analyzed during the current study are available from the corresponding authors on reasonable request.

## Abbreviations

KAP: Knowledge, Attitudes, and Practices
Hb: Hemoglobin
SEA: Southeast Asia
LCH: Laem Chabang Hospital
IQR: Inter-quartile ranges
SAS: Statistical Analysis Software
NC: North Carolina
USA: United States of America
CBC: Complete blood count
HPLC: High-performance liquid chromatography
PCR: Polymerase chain reaction
NA: Not available
CI: 95% confidence interval
ASEAN: Association of Southeast Asian Nations
NT: Not tested
CS: Constant Spring
PS: Pakse
QZ: Quang Sze
WM: Westmead
