# Supplementary material for: Feasibility of and barriers to thalassemia screening in migrant populations: a cross-sectional study of Myanmar and Cambodian migrants in Thailand
Source: BMC Public Health. 2021 Jun 21;21:1177. doi: 10.1186/s12889-021-11059-2 (PMC8215823; doi:10.1186/s12889-021-11059-2)
Supplement: Supplementary file 1 — Additional file 1. Survey tools for thalassemia, Demographic and KAP surveys developed for this study are provided in English, Thai, Burmese, and Khmer languages. [file 12889_2021_11059_MOESM1_ESM.docx]

**SUPPLEMENTARY MATERIALS**

**Supplemental Tables:**

**Supplemental Table 1.** Carrier rates (in %) for common hemoglobin disorders in Southeast Asia.^†^

| **Country** | **α^0^-thalassemia** | **α^+^-thalassemia** | **β-thalassemia** | **Hemoglobin E** |
| --- | --- | --- | --- | --- |
| Brunei (1, 2) | 0 | 4.3 | 2–22.7 | 0–3.7 |
| Cambodia | 1 | 15.5 | 2.8 | 10–54 |
| Indonesia | <1 | 3–20 | 3 | 1–33 |
| Laos (3, 4) | 8.7–13.9 | 11–17.7 | 3.5–5 | 22.9– >30 |
| Malaysia | 4.5 | 16 | 4.5 | 1–3 |
| Myanmar | NA | 10 | 1–5.3 | 4–48 |
| Philippines | 5 | 2.2 | 1 | NA |
| Singapore | 2–3 | 1–3 | 0.93 | 0.64 |
| Thailand | 2.2–9 | 8–30 | 1–3 | 10–50 |
| Vietnam | NA | 3.5 | 4 | 10–20 |

NA = not available.

^†^ Adapted from Viprakasit et al., 2009.(5)

**Supplemental Table 2.** Common α- and β-globin mutations identified using previously described multiplex PCR-based techniques.(6)

| **No.** | **Globin gene type** | **Mutation** | **Mutation type** |
| --- | --- | --- | --- |
| 1 | α-globin | -α^3.7^ | Single deletion |
| 2 |  | -α^4.2^ | Single deletion |
| 3 |  | - -^SEA^ | Double deletion |
| 4 |  | - -^THAI^ | Double deletion |
| 5 |  | - -^FIL^ | Double deletion |
| 6 |  | - -^MED^ | Double deletion |
| 7 |  | -(α)^20.5^ | Double deletion |
| 8 |  | - -^SIAM^ | Double deletion^†^ |
| 9 |  | Initiation codon (ATG>A-G) | Non-deletion |
| 10 |  | Codon 30 (∆GAG) | Non-deletion |
| 11 |  | Codon 59 (GGC > GAC) | Non-deletion |
| 12 |  | Codon 142 (Pakse; TAA > TAT) | Non-deletion |
| 13 |  | Codon 125 (Quang Sze; CTG > CCG) | Non-deletion |
| 14 |  | Codon 142 (Constant Spring; TAA > CAA) | Non-deletion |
| 15 | β-globin | nt -28 (A > G) | β^+^ |
| 16 |  | Codon 8/9 (+G) | β^0^ |
| 17 |  | IVSI-5 (G > C) | β^+^ |
| 18 |  | Codon 41/42 (-TTCT) | β^0^ |
| 19 |  | Codon 71/72 (+A) | β^0^ |
| 20 |  | Codon 17 (A >T) | β^0^ |
| 21 |  | IVSI-1 (G > T) | β^0^ |
| 22 |  | Codon 26 Hb E (GAG > AAG) | β^E^ |
| 23 |  | IVSII-654 (C > T) | β^+^ |
| 24 |  | Codon 35 (C >A) | β^0^ |
| 25 |  | Codon 43 (G >T) | β^0^ |
| 26 |  | Codon 26 (G >T) | β^0^ |
| 27 |  | Codon 95 (+A) | β^0^ |
| 28 |  | Codon 19 (A>G) | β^+^ |
| 29 |  | Codon 41 (-C) | β^0^ |
| 30 |  | Codon 27/28 (+C) | β^0^ |

^†^ The α-globin deletion - -^SIAM^ was identified using the methodology described in Riolueang et al. 2019.(7)

**Supplemental Figures:**

**Supplemental Figure 1.** CONSORT flow diagram for surveys and thalassemia testing (the latter in gray).





Thai subjects presenting to Laem Chabang Hospital (LCH) for outpatient care and migrant (Myanmar and Cambodian) subjects presenting to LCH for health registration were screened for eligibility. Those pregnant or with a pregnant partner by self-report were excluded. A total of 200 Thai and 300 migrant subjects were surveyed. The KAP survey was then completed by 155 Thai and 11 migrants who were aware of thalassemia. Out of the 300 migrant subjects offered thalassemia testing, 286 were eligible for and 277 (96.9%) consented to thalassemia testing.

**Supplemental Figure 2A.** The demographic survey developed for the study of thalassemia in English.

| DEMOGRAPHIC SURVEY: 1. Age: _________ years  2. Gender: Male Female  3. Nationality: Thai Migrant  4. Country of origin (if migrant): Myanmar Cambodia Other ________________ |
| --- |
| 5. Primary language (choose 1): Thai Burmese Khmer English  Other ______________  6. Additional languages (choose any): Thai Burmese Khmer English  Other ______________  7. Educational level: None Primary (1-6) Junior high (7-9) Senior high (10-12)  Higher education_________________  8. Work sector: Fishery Manufacturing Domestic Construction  Other ________________  9. Duration of time living in Thailand (if migrant): __________ years ___________ months  10. Number of times moved to a different city within Thailand (if migrant): __________  (“0” if only lived in Laem Chabang)  11. First time participating in health registration? (if migrant): First time Renewal  12. How did you pay for your health care before today?: Employer health insurance  Public health insurance Migrant health insurance scheme Private health insurance  Cash Other______________  13. Where do you go *first* when you are sick?: Drug store Doctor Traditional healer  Other_________  14. What is the number of times that you have visited a doctor or a hospital in the prior 1 year?:  __________  15. Marital status: Single Married Divorced  16. Nationality of partner (if Married): Thai Migrant  17. Country of origin of partner (if Married): Myanmar Cambodia Other __________  18. Number of children: __________  19. Have you ever heard of anemia?: Yes No  20. Have you ever heard of thalassemia?: Yes No  Have you ever had:  21. Anemia: Yes No  22. Thalassemia: Yes No  23. Transfusions: Yes No  24. Miscarriages: Yes No  Has anyone in your family ever had:  25. Anemia: Yes No  26. Thalassemia: Yes No  27. Transfusions: Yes No  28. Miscarriages: Yes No |
| (For those subjects who answered “No” to Question 20, please answer Questions 29-32):  29. Would you want to know if you were a carrier for a disease that you could pass on to your child before you have a baby? Would want to know Would not want to know  30. If you knew you were a carrier for a disease that you could pass on to your child, would you want to have your baby tested? Would want to test Would not want to test  31. If you knew you were a carrier for a disease that you could pass on to your child, would you want your partner to be tested? Would want to test Would not want to test  32. What is the best way to give you more information about a medical condition like thalassemia?:  Consultation with doctor Brochure Company-sponsored workshop  Government-sponsored workshop Other________ |

**Supplemental Figure 2B.** The demographic survey developed for the study of thalassemia in Thai.

| การสำรวจข้อมูลประชากร: 1. อายุ: _________ ปี  2. เพศ: ชาย หญิง  3. สัญชาติ: ไทย ต่างชาติ  4. ประเทศบ้านเกิด (ชาวต่างชาติ):  **พม่า**  **ลาว**  **อื่นๆ** ________________ |
| --- |
| 5. ภาษาหลัก (เลือก 1 ข้อ):  **ภาษาไทย**  **ภาษาพม่า**  **ภาษาลาว**  **ภาษาอังกฤษ อื่นๆ** ____________  6. ภาษารอง (เลือกได้มากกว่า 1 ข้อ):  **ภาษาไทย**  **ภาษาพม่า**  **ภาษาลาว**  **ภาษาอังกฤษ อื่นๆ** ____________  7. การศึกษา:  **ไม่ได้ศึกษา**  **ประถมศึกษา**  **มัธยมศึกษาตอนต้น**  **มัธยมศึกษาตอนปลาย**  สูงกว่ามัธยมศึกษาตอนปลาย ระบุ____________________  8. ภาคการจ้างงาน:  **การประมง**  **อุตสาหกรรม**  **แม่บ้าน** การก่อสร้าง อื่นๆ ________________  9. ระยะเวลาที่อาศัยอยู่ในประเทศไทย (ชาวต่างชาติ): ______ปี _______เดือน  10. จำนวนครั้งที่ย้ายไปอาศัยอยู่ที่เมืองต่างๆ ในประเทศไทย (ชาวต่างชาติ): __________ครั้ง (ตอบ 0 หากอาศัยอยู่เพียงที่แหลมฉบัง)  11. เป็นครั้งแรกที่เข้าร่วมการลงทะเบียนประกันสุขภาพของท่านหรือไม่? (ชาวต่างชาติ): ครั้งแรก ต่ออายุ  12. ท่านมีหลักประกันสุขภาพอะไรบ้าง? หรือมีวิธีการชำระค่ารักษาพยาบาลอย่างไรบ้าง?:  **การประกันสุขภาพจากนายจ้าง** หลักประกันสุขภาพจากรัฐบาล  **การประกันสุขภาพจากสำหรับผู้อพยพ**  **การประกันสุขภาพส่วนบุคคล**  เงินสด อื่นๆ_____________________  13. เมื่อท่านเจ็บป่วย **สถานที่/บุคคลแรก** ที่ท่านจะไปรับการรักษา คือ :  **ร้านขายยา** แพทย์แผนปัจจุบัน  **แพทย์แผนโบราณ อื่นๆ____________**  14. ในช่วง 1 ปีที่ผ่านมา ท่านเคยไปพบแพทย์ หรือไปโรงพยาบาลมาแล้วกี่ครั้ง?: __________  15. สถานภาพ:  **โสด**  **แต่งงาน**  **หย่าร้าง**  16. หาก **สมรส**: สัญชาติของคู่สมรส:  **ไทย**  **ต่างชาติ**  17. ประเทศบ้านเกิดของ**คู่สมรส**:  **พม่า**  **ลาว**  **อื่นๆ** ________________  18. จำนวนบุตร: __________คน  19. ท่านเคยได้ยินเกี่ยวกับโรคโลหิตจาง/ซีด หรือไม่?:  **เคย**  **ไม่เคย**  20. ท่านเคยได้ยินเกี่ยวกับโรคธาลัสซีเมียหรือไม่?: เคย  **ไม่เคย** |
| ท่านมีอาการหรือภาวะเหล่านี้ใช่หรือไม่?:  21. โลหิตจาง/ซีด: ใช่ ไม่ใช่  22. ธาลัสซีเมีย: ใช่ ไม่ใช่  23. การให้เลือด: ใช่ ไม่ใช่  24. การแท้ง: ใช่ ไม่ใช่  มีคนในครอบครัวของท่านมีอาการหรือภาวะเหล่านี้ ใช่หรือไม่?:  25. โลหิตจาง/ซีด: ใช่ ไม่ใช่  26. ธาลัสซีเมีย: ใช่ ไม่ใช่  27. การให้เลือด: ใช่ ไม่ใช่  28. การแท้ง: ใช่ ไม่ใช่  *(ในกรณีที่ท่านตอบ* ***“ไม่เคย”*** *ใน* ***ข้อ 20*** *กรุณาตอบคำถามข้อ 29-32)*  29. ท่านต้องการทราบหรือไม่ว่า ตัวท่านเองเป็นพาหะของโรคใดๆ ที่สามารถถ่ายทอดไปสู่ลูกของท่านได้ ก่อนที่ท่านจะมีลูก?  ต้องการทราบ ไม่ต้องการทราบ  30. หากท่านทราบแล้วว่า ตัวท่านเป็นพาหะของโรคใดๆ ที่สามารถถ่ายทอดไปสู่ลูกของท่านได้ ท่านจะต้องการให้ลูกของท่านได้รับการตรวจด้วยหรือไม่? ต้องการให้ตรวจ ไม่ต้องการให้ตรวจ  31. หากท่านทราบแล้วว่าตัวท่านเป็นพาหะของโรคใดๆ ที่สามารถถ่ายทอดไปสู่ลูกของท่านได้ ท่านจะต้องการให้คู่สมรสของท่านได้รับการตรวจด้วยหรือไม่? ต้องการให้ตรวจ ไม่ต้องการให้ตรวจ   32. ท่านคิดว่ามีวิธีการใดบ้างที่จะทำให้ท่านเข้าถึงข้อมูลความรู้เกี่ยวกับโรคธาลัสซีเมีย?: ปรึกษาแพทย์  **เอกสารแผ่นพับ**  **โครงการอบรมเพิ่มความรู้จากภาคเอกชน โครงการอบรมเพิ่มความรู้จากรัฐบาล อื่นๆ __________________** |

**Supplemental Figure 2C.** The demographic survey developed for the study of thalassemia in Burmese.

| စာရင္းေကာက္ေသာ ေယဘုယ် သံု ုးသပ္ခ်က္ -  ၁. အသက္ - ႏ ွစ္  ၂. လိင္ -  က်ား  မ  ၃. လူမ်ဳိး -  ထိုင္း  ေရႊ႕ေျပာင္းေနထိုင္သူ  ၄. မူလႏိုင္ငံ (ေရႊ႕ေျပာင္းေနထိုင္သူျဖစ္လွ်င္) -  ျမန္မာ  လာအို  အျခား |
| --- |
| ၅. မူရင္း ဘာသာစကား (တစ္မ်ဳိးေရြးပါ) -  ထိုင္း  ျမန္မာ  လာအို  အဂၤလိပ္  အျခား  ၆. အပို ဘာသာစကား (ႀကိဳက္ရာေရြးပါ) -  ထိုင္း  ျမန္မာ  လာအို  အဂၤလိပ္  အျခား  ၇. ပညာအရည္အခ်င္း -  မရွိ  မူလတန္း (၁-၆)  အလယ္တန္း (၇-၉)  အထက္တန္း (10-12)   တကၠသိုလ္  ၈. လုပ္ငန္းခြင္ -  ငါးလုပ္ငန္း  ထုတ္လုပ္မႈလုပ္ငန္း  ျပည္တြင္းလုပ္ငန္း  ေဆာက္လုပ္ေရးလုပ္ငန္း  အျခား |
| ၉. ထိုင္းႏိုင္ငံတြင္ ေနထိုင္ေသာ ၾကာခ်ိန္ (ေရႊ႕ေျပာင္းေနထိုင္သူျဖစ္လွ်င္) - ႏ ွစ္ လ  ၁၀. ထိုင္းႏိုင္ငံအတြင္း ကြဲျပားေသာ ၿမိဳ႕မ်ားသို႔ ေရႊ႕ေျပာင္းခဲ့သည့္ အႀကိမ္အေရအတြက္ (ေရႊ႕ေျပာင္းေနထိုင္သူျဖစ္လွ်င္) - |
| (“0” သည္ Laem Chabang တြင္သာ ေနထိုင္ခဲ့လွ်င္)  ၁၁. က်န္းမာေရးဆိုင္ရာ မွတ္ပံုတင္ျခင္းတြင္ ပထမအႀကိမ္ ပါ၀င္ျခင္း (ေရႊ႕ေျပာင္းေနထိုင္သူျဖစ္လွ်င္) -  ပထမအႀကိမ္   သက္တမ္းတိုးျခင္း  ၁၂. ယေန႔ မတိုင္ခင္က က်န္းမာေရး ေစာင့္ေရွာက္မႈအတြက္ ဘယ္လို ေငြေပးေခ်ခဲ့သလဲ -  အလုပ္ရွင္မွ က်န္းမာေရး အာမခံ  ျပည္သူ႔ က်န္းမာေရး အာမခံ  ေရႊ႕ေျပာင္းေနထိုင္သူ က်န္းမာေရး အာမခံ  ပုဂၢလိက က်န္းမာေရး အာမခံ  ေငြ သား  အျခား  ၁၃. ဖ်ားနာသည့္အခါ ပထမဆံုး ဘယ္ေနရာသို႔ သြားသလဲ -  ေဆးဆိုင္  ဆရာ၀န္  တိုင္းရင္းေဆးဆရာ  အျခား |
| ၁၄. လြန္ခဲ့ေသာ ၁ ႏွစ္က ေဆးရံု သို႔မဟုတ္ ဆ ရာ၀န္ဆီ ဘယ္ႏွစ္ႀကိမ္ သြားခဲ့သလဲ -  ၁၅. အိမ္ေထာင္ ရွိ / မရွိ -  လူလြတ္  အိမ္ေထာင္ရွိ  ကြာရွင္း  ၁၆. အိမ္ေထာင္ဘက္၏ လူမ်ဳိး (အိမ္ေထာင္ရွိလွ်င္) -  ထိုင္း  ေရႊ႕ေျပာင္းေနထိုင္သူ  ၁၇. အိမ္ေထာင္ဘက္၏ မူလႏိုင္ငံ (အိမ္ေထာင္ရွိလွ်င္) -  ျမန္မာ  လာအို  အျခား  ၁၈. ကေလးအေရအတြက္ -  ၁၉. ေသြးအားနည္းေရာဂါအေၾကာင္း ၾကားဖူးပါသလား -  ၾကားဖူးပါသည္  မၾကားဖူးပါ  ၂၀. ေသြးအားနည္းေရာဂါအေၾကာင္း ၾကားဖူးပါသလား -  ၾကားဖူးပါသည္  မၾကားဖူးပါ  သင့္မွာ ရွိခဲ့ဖူးသလား -  ၂၁. ေသြးအားနည္းျခင္း -  ရွိ  မရွိ  ၂၂. ေသြးအားနည္းေရာဂါ -  ရွိ  မရွိ  ၂၃. ေသြးသြင္းျခင္း -  ရွိ  မရွိ  ၂၄. ကိုယ္၀န္ပ်က္ျခင္း -  ရွိ  မရွိ  သင္၏ မိသားစုတြင္ တစ္စံုတစ္ေယာက္ ရွိခဲ့ဖူးသလား -  ၂၅. ေသြးအားနည္းျခင္း -  ရွိ  မရွိ  ၂၆. ေသြးအားနည္းေရာဂါ -  ရွိ  မရွိ  ၂၇. ေသြးသြင္းျခင္း -  ရွိ  မရွိ  ၂၈. ကိုယ္၀န္ပ်က္ျခင္း -  ရွိ  မရွိ |
| (ေမးခြန္း နံပါတ္ ၂၀ အား “မရ”ွိ ဟု ေျဖခဲ့သူမ်ားအတြက္ ေမးခြန္း ၂၉-၃၂ အား ေျဖဆိုေပးပါ။) -  ၂၉. ကေလး မရခင္က သင့္ကေလးဆီသို႔ လက္ဆင့္ကမ္းႏုိင္သည့္ ေရာဂါ သယ္ေဆာင္သူျဖစ္ခဲ့လွ်င္ သင္သိလိုပါသလား။   သိလိုပါသည္  မသိလိုပါ  ၃၀. သင့္ကေလးဆီသို႔ လက္ဆင့္ကမ္းႏုိင္သည့္ ေရာဂါ သယ္ေဆာင္သူအျဖစ္ သိခဲ့လွ်င္ သင့္ကေလးအား စစ္ေဆးစမ္းသပ္ လိုပါ သလား။  စမ္းသပ္လိုပါသည္  မစမ္းသပ္လိုပါ  ၃၁. သင့္ကေလးဆီသို႔ လက္ဆင့္ကမ္းႏုိင္သည့္ ေရာဂါ သယ္ေဆာင္သူအျဖစ္ သိခဲ့လွ်င္ သင့္အိမ္ေထာင္ဘက္အား စစ္ေဆးစမ္းသပ္ လုိပါသလား။  စမ္းသပ္လိုပါသည္  မစမ္းသပ္လိုပါ  ၃၂. ေသြးအားနည္းေရာဂါကဲ့သို႔ ေဆးပညာရပ္ဆိုင္ရာ အေျခအေနအေၾကာင္း သတင္းပိုေပးႏုိင္ရန္ မည္သည့္ နည္းလမ္းသည္ အေ ကာင္းဆံုး ျဖစ္သနည္း။   ဆရာ၀န္ႏွင့္တိုင္ပင္ေဆြးေႏြးျခင္း  လက္ကမ္းေၾကျငာစာအုပ္ငယ္  ကုမၸဏီမွ အကုန္အက်ခံေသာ အလုပ္ရံုေဆြးေႏြးပြဲ  အစိုးရမွ အကုန္အက်ခံေသာ အလုပ္ရံုေဆြးေႏြးပြဲ  အျခား |

**Supplemental Figure 2D.** The demographic survey developed for the study of thalassemia in Khmer.

ការស្ទង់មតិប្រជាសាស្រ្ត:

1. អាយុ:______ឆ្នាំ
2. ភេទ: □ ប្រុស □ស្រី
3. សញ្ជាតិ: □ ថៃ □ ជនចំណាកស្រុក
4. មកពីប្រទេស(ប្រសិនជាជនចំណាកស្រុក): □ ភូមា □ឡាវ □ ផ្សេងៗ_____________

5. ភាសាបឋម (ជ្រើសរើសយក 1): □ថៃ □ភូមា □ឡាវ □ភាសាអង់គ្លេស

□ផ្សេងទៀត ______________

6. ភាសាបន្ថែម (ជ្រើសរើសណាមួយ): □ថៃ □ភូមា □ឡាវ □អង់គ្លេស

                                     □ផ្សេងទៀត ______________

7. កម្រិតអប់រំ: □គ្មាន □ថ្នាក់បឋមសិក្សា (1-6) □ថ្នាក់វិទ្យាល័យ (7-9)

□ជាន់ខ្ពស់ខ្ពស់ (10-12)  □ការអប់រំខ្ពស់ _________________________

8. វិស័យ: □វិស័យជលផល □ផលិត □ក្នុងស្រុក □សំណង់ □ ផ្សេងៗ ________________

9. រយៈពេលនៃការរស់នៅក្នុងប្រទេសថៃ (ប្រសិនបើអ្នកចំណាកស្រុក): __________ ឆ្នាំ ___________ ខែ

10. ចំនួនដងបានផ្លាស់ទីលំនៅទៅទីក្រុងផ្សេងគ្នាក្នុងប្រទេសថៃ (ប្រសិនបើជនអន្តោប្រវេសន៍): __________ ("0" ប្រសិនបើរស់នៅតែនៅក្នុងទីក្រុង Laem Chabang)

11. ជាលើកដំបូងចូលរួមក្នុងការចុះឈ្មោះសុខភាព? (ប្រសិនបើអន្តោរប្រវេសន៍):

□ពេលវេលាដំបូង □បន្តឡើងវិញ

12. តើអ្នកបានបង់ប្រាក់សម្រាប់ការថែទាំសុខភាពរបស់អ្នកយ៉ាងដូចម្ដេចមុនថ្ងៃនេះ ?:

□ការធានារ៉ាប់រងសុខភាពរបស់និយោជក □ការធានារ៉ាប់រងសុខភាពសាធារណៈ

□គម្រោងធានារ៉ាប់រងសុខភាពអន្តោប្រវេសន៍ □ការធានារ៉ាប់រងសុខភាពឯកជន □សាច់ប្រាក់ □ផ្សេងៗ ______

13. តើអ្នកទៅទីណានៅពេលអ្នកឈឺ? □ហាងឱសថ □វេជ្ជបណ្ឌិត □អ្នកព្យាបាលជំងឺបុរាណ

□ផ្សេងទៀត _________

14. តើចំនួនដងដែលអ្នកបានទៅជួបគ្រូពេទ្យឬមន្ទីរពេទ្យក្នុងរយៈពេល 1 ឆ្នាំមុន?:

__________

15. ស្ថានភាពគ្រួសារ: □សំលៀកបំពាក់ □រៀបការ □លះលែង

16. សញ្ជាតិរបស់ដៃគូរ (ប្រសិនបើរៀបការ): □ថៃ □អ្នកចំណាកស្រុក

17.ប្រទេសដែលជាដៃគូ (បើរៀបការ): □ប្រទេសមីយ៉ាន់ម៉ាឡាវឡាវ

□ផ្សេងទៀត ________________

18.ចំនួនកុមារ: __________

19.តើអ្នកធ្លាប់ឮអំពីជំងឺខ្វះឈាមក្រហមដែរឬទេ? □បាទ/ចាស □ទេ

20. តើអ្នកធ្លាប់លឺអំពីជំងឺ Thalassemia? □បាទ/ចាស □ទេ

តើអ្នកធ្លាប់មាន:

21. ភាពស្លេកស្លាំង: □បាទ/ចាស □ទេ

22. Thalassemia : □បាទ/ចាស □ទេ

23. ការបញ្ជូនសារ: □បាទ/ចាស □ទេ

24.ការរលូតកូន: □បាទ/ចាស □ទេ

មាននរណាម្នាក់នៅក្នុងគ្រួសាររបស់អ្នកធ្លាប់មាន:

25. ភាពស្លកសាំង: □បាទ/ចាស □ទេ

26. Thalassemia: □បាទ/ចាស □ទេ

27. ការបញ្ជូនចូល: □បាទ/ចាស □ទេ

28.ការរលូតកូន: □បាទ/ចាស □ទេ

(ចំពោះអ្នកដែលឆ្លើយថា "ទេ" ចំពោះសំណួរទី 20 សូមឆ្លើយសំណួរទី 29-32):

29. តើអ្នកចង់ដឹងថាអ្នកជាអ្នកផ្ទុកជំងឺអ្វីដែលអ្នកអាចបញ្ជូនទៅកូនរបស់អ្នកមុនពេលអ្នកមានកូនឬទេ? □ចង់ដឹង □មិនចង់ដឹងទេ

30. ប្រសិនបើអ្នកដឹងថាអ្នកជាអ្នកជម្ងឺដែលអ្នកអាចបញ្ជូនទៅកូនអ្នកតើអ្នកចង់ឱ្យទារករបស់អ្នកត្រូវបានធ្វើតេស្តឬទេ? □ចង់សាកល្បង □មិនចង់សាកល្បង

31. ប្រសិនបើអ្នកដឹងថាអ្នកជាអ្នកជម្ងឺដែលអ្នកអាចបញ្ជូនទៅកូនអ្នកតើអ្នកចង់ឱ្យដៃគូរបស់អ្នកត្រូវបានធ្វើតេស្តឬទេ? □ចង់សាកល្បង □មិនចង់សាកល្បង

32. តើអ្វីជាមធ្យោបាយដ៏ល្អបំផុតដើម្បីផ្តល់ព័ត៌មានបន្ថែមអំពីស្ថានភាពសុខភាពដូចជាជំងឺThalassemia ?:

      □ពិគ្រោះជាមួយវេជ្ជបណ្ឌិត □ខិត្តប័ណ្ណ □សិក្ខាសាលាដែលឧបត្ថម្ភដោយក្រុមហ៊ុន

      □សិក្ខាសាលាដែលឧបត្ថម្ភដោយរដ្ឋាភិបាល □ផ្សេងៗ________

**Supplemental Figure 3A.** The Knowledge, Attitudes, and Practices (KAP) survey developed for the study of thalassemia in English.

KAP Survey:

Knowledge (yes/no)

1. Thalassemia is a disease of the blood.
2. Thalassemia is one of the causes of anemia.
3. Thalassemia is a rare condition in Asia.
4. Thalassemia is an infectious disease, meaning you can catch it from someone else.
5. Thalassemia is an inherited disease, meaning parents can pass it on to their children.
6. Even if the parents do not have thalassemia, their child could still get thalassemia.
7. If two parents have a child with thalassemia, they are at risk for having another child with thalassemia later on.
8. A blood test can be done to see if someone has thalassemia.
9. A thalassemia carrier requires regular blood transfusions.
10. People with thalassemia major require regular blood transfusions.
11. A thalassemia carrier can eventually develop thalassemia major.
12. Thalassemia can be cured with a pill.

Attitudes towards severe thalassemia (Likert scale for positive items: strongly agree = 5;

agree = 4; unsure = 3; disagree = 2; strongly disagree = 1; reverse for negative items)

*First explain that a child with severe thalassemia is likely to be sick frequently and suffer from the disease.*

1. Thalassemia should be prevented in the general population.
2. Having a child with thalassemia is a burden.
3. Having a child with thalassemia is a blessing (i.e. can be considered lucky).
4. I would want to know if I were having a baby with thalassemia.
5. I would get tested during pregnancy if thalassemia testing is available.
6. I would want my partner to get tested during pregnancy if thalassemia testing is available.
7. I would continue having children with my partner if we were both thalassemia carriers.
8. I would not want to know if my baby was thalassemic.
9. Testing for thalassemia is not useful to me during pregnancy.
10. I would not have more children with my partner if we were both thalassemia carriers.
11. I would end a pregnancy if the baby has thalassemia.
12. I disagree with ending a pregnancy.
13. I would not prevent a thalassemic baby from being born.
14. It would be better to end a pregnancy with a thalassemic baby than to let the child be born and suffer.

Practices (Yes/No)

1. I have been tested for thalassemia before.
2. My partner has been tested for thalassemia before.
3. (If “Yes” to either Question 27 or 28) I or my partner have had prenatal diagnosis (PND) to find out if the baby was affected by thalassemia.
4. (If “Yes” to Question 29) Did the PND show that your baby had thalassemia:
5. I plan to have 1 or more child in the future.

**Supplemental Figure 3B.** The Knowledge, Attitudes, and Practices (KAP) survey developed for the study of thalassemia in Thai.

# การสำรวจความคิดเห็น:

**ความรู้ ความเข้าใจ ต่อโรคธาลัสซีเมีย** (ตอบ **ใช่** หรือ **ไม่ใช่**)

| **คำถาม** | **คำตอบ** | |
| --- | --- | --- |
| 1. โรคธาลัสซีเมียเป็นโรคเลือดชนิดหนึ่ง ใช่หรือไม่? | ใช่ | ไม่ใช่ |
| 2. โรคธาลัสซีเมียเป็นสาเหตุของโลหิตจาง หรือซีด ใช่หรือไม่? | ใช่ | ไม่ใช่ |
| 3. โรคธาลัสซีเมียพบได้ยากในทวีปเอเชีย ใช่หรือไม่? | ใช่ | ไม่ใช่ |
| 4. โรคธาลัสซีเมียเป็นโรคติดต่อ ใช่หรือไม่? | ใช่ | ไม่ใช่ |
| 5. โรคธาลัสซีเมียเป็นโรคพันธุกรรมที่ถ่ายทอดจากพ่อแม่สู่ลูก ใช่หรือไม่? | ใช่ | ไม่ใช่ |
| 6. ถึงแม้พ่อแม่จะไม่ได้เป็นโรคธาลัสซีเมีย แต่ลูกก็สามารถเป็นโรคธาลัสซีเมียได้ ใช่หรือไม่? | ใช่ | ไม่ใช่ |
| 7. ถ้าพ่อแม่มีลูก 2 คนแรกเป็นโรคธาลัสซีเมียแล้ว มีโอกาสสูงที่ลูกคนถัดไปจะเป็นโรคธาลัสซีเมียด้วยเช่นกัน ใช่หรือไม่? | ใช่ | ไม่ใช่ |
| 8. การตรวจเลือด เป็นวิธีที่จะทำให้ทราบว่าบุคคลใดเป็นโรคธาลัสซีเมีย ใช่หรือไม่? | ใช่ | ไม่ใช่ |
| 9. ผู้ที่เป็นพาหะโรคธาลัสซีเมียต้องได้รับการให้เลือด ใช่หรือไม่? | ใช่ | ไม่ใช่ |
| 10. ผู้ที่เป็นโรคธาลัสซีเมียชนิดรุนแรงต้องได้รับการให้เลือด ใช่หรือไม่? | ใช่ | ไม่ใช่ |
| 11. ท่านคิดว่าหากท่านเป็นพาหะโรคธาลัสซีเมียแล้ว ท่านจะกลายเป็นโรคธาลัสซีเมียชนิดรุนแรงต่อไปได้ ใช่หรือไม่? | ใช่ | ไม่ใช่ |
| 12. โรคธาลัสซีเมียสามารถรักษาให้หายขาดได้โดยการรับประทานยา ใช่หรือไม่? | ใช่ | ไม่ใช่ |

**ทัศนคติที่มีต่อโรคธาลัสซีเมีย**

เป็นความคิดเห็นของท่านต่อความเจ็บป่วยของเด็กที่เป็นโรคธาลัสซีเมีย

(ให้คะแนนตามลำดับความเห็นด้วย ได้แก่ เห็นด้วยอย่างยิ่ง = 5, เห็นด้วย = 4, ไม่แน่ใจ = 3, ไม่เห็นด้วย = 2, ไม่เห็นด้วยอย่างยิ่ง = 1

| **ความคิดเห็น** | **ระดับคะแนน** | | | | |
| --- | --- | --- | --- | --- | --- |
|  | **5** | **4** | **3** | **2** | **1** |
| 13. เราต้องป้องกันการเกิดโรคธาลัสซีเมียในคนทั่วไป |  |  |  |  |  |
| 14. การมีลูกเป็นโรคธาลัสซีเมียถือเป็นภาระของท่าน |  |  |  |  |  |
| 15. ท่านเชื่อว่าการมีลูกเป็นโรคธาลัสซีเมียถือเป็นโชคดีของท่าน |  |  |  |  |  |
| 16. ท่านอยากจะทราบว่าท่านจะมีลูกที่เป็นโรคธาลัสซีเมียหรือไม่ |  |  |  |  |  |
| 17. ท่านต้องการตรวจโรคธาลัสซีเมียระหว่างตั้งครรภ์ |  |  |  |  |  |
| 18. ท่านต้องการให้คู่สมรสของท่านตรวจโรคธาลัสซีเมียระหว่างตั้งครรภ์ด้วย |  |  |  |  |  |
| 19. ถึงแม้ท่านและคู่สมรสของท่านเป็นพาหะโรคธาลัสซีเมียทั้งคู่ ท่านก็ยังต้องการมีลูกต่อไป |  |  |  |  |  |
| 20. ท่านไม่ต้องการทราบว่าลูกของท่านเป็นโรคธาลัสซีเมีย |  |  |  |  |  |
| 21. ท่านคิดว่าการตรวจโรคธาลัสซีเมียในระหว่างตั้งครรภ์ไม่เป็นประโยชน์สำหรับตัวท่าน |  |  |  |  |  |
| 22. ท่านไม่ต้องการมีลูกกับคู่สมรสของท่าน หากท่านทราบว่าท่านทั้งคู่ต่างเป็นพาหะโรคธาลัสซีเมีย |  |  |  |  |  |
| 23. ท่านจะยุติการตั้งครรภ์หากทราบว่าลูกของท่านเป็นโรคธาลัสซีเมีย |  |  |  |  |  |
| 24. ท่านไม่เห็นด้วยที่จะให้มีการยุติการตั้งครรภ์ |  |  |  |  |  |
| 25. ท่านจะไม่ยุติการตั้งครรภ์ แม้ทราบว่าทารกในครรภ์ของท่านเป็นโรคธาลัสซีเมีย |  |  |  |  |  |
| 26. ท่านเห็นว่าการยุติการตั้งครรภ์ของทารกที่เป็นโรคธาลัสซีเมียเป็นวิธีที่ดีกว่าการให้ทารก  คลอดออกมาแล้วเกิดการเจ็บป่วยและทุกข์ทรมานจากการเจ็บป่วยภายหลัง |  |  |  |  |  |

**การตรวจคัดกรองโรคธาลัสซีเมีย** (ตอบ **ใช่** หรือ **ไม่ใช่**)

| **คำถาม** | **คำตอบ** | |
| --- | --- | --- |
| 27. ท่านเคยตรวจคัดกรองโรคธาลัสซีเมียแล้ว ใช่หรือไม่? | ใช่ | ไม่ใช่ |
| 28. คู่สมรสของท่านเคยตรวจคัดกรองโรคธาลัสซีเมียแล้ว ใช่หรือไม่? | ใช่ | ไม่ใช่ |
| 29. (กรณีตอบ **“ใช่”** จากคำถามข้อ 27 และ/หรือ 28) ท่านหรือคู่สมรสของท่านได้ตรวจวินิจฉัยโรคธาลัสซีเมียของทารกในครรภ์ก่อนคลอดด้วย ใช่หรือไม่? | ใช่ | ไม่ใช่ |
| 30. (กรณีตอบ **“ใช่”** จากคำถามข้อ 29) ทารกของท่านเป็นโรคธาลัสซีเมีย ใช่หรือไม่? | ใช่ | ไม่ใช่ |
| 31. ท่านวางแผนจะมีลูกมากกว่า 1 คน ใช่หรือไม่? | ใช่ | ไม่ใช่ |

**Supplemental Figure 3C.** The Knowledge, Attitudes, and Practices (KAP) survey developed for the study of thalassemia in Burmese.

KAP SURVEY:

ဗဟုသုတ (မွန္ / မွား)

1. ေသြးအားနည္းေရာဂါသည္ ေသြးေရာဂါ တစ္ခု ျဖစ္သည္။

2. ေသြးအားနည္းေရာဂါသည္ ေသြးအားနည္းျခင္းေၾကာင့္ ျဖစ္သည္။

3. ေသြးအားနည္းေရာဂါသည္ အာရွတြင္ ျဖစ္ခဲေသာ အေျခအေန ျဖစ္သည္။

4. ေသြးအားနည္းေရာဂါသည္ တစ္စံု တစ္ေယာက္ဆီမွ ကူစက္ႏိုင္သည့္ ကူးစက္ေရာဂါ တစ္မ်ဳိး ျဖစ္သည္။

5. ေသြးအားနည္းေရာဂါသည္ မိဘဆီမွ သားသမီးဆီသို႔ သယ္ေဆာင္ႏိုင္သည့္ အေမြဆက္ခံႏိုင္ေသာ ေရာဂါတစ္မ်ဳိး ျဖစ္သည္။

6. မိဘမ်ားတြင္ ေသြးအားနည္းေရာဂါ မရွိသည့္တိုင္ေအာင္ ၎တုိ႔၏ သားသမီးမ်ားတြင္ ေသြးအားနည္းေရာဂါ ရႏိုင္သ

ည္။

7. မိဘႏွစ္ပါးတြင္ ေသြးအားနည္းေရာဂါသည္ ကေလးရွိလွ်င္ ေနာက္ပိုင္းတြင္ ေနာက္ကေလးတြင္ ေသြးအားနည္းေရာဂါ ရွိရန္အတြက

ျဖစ္ႏိုင္ေျခ ရိႈသည္။

8. တစ္စံုတစ္ေယာက္တြင္ ေသြးအားနည္းေရာဂါရွိခဲ့လွ်င္ ေသြးစစ္ျခင္းျဖင့္ သိႏိုင္သည္။

9. ေသြးအားနည္းေရာဂါ ရွိသူသည္ ပံုမွန္ ေသြးလဲေပးရန္ လိုအပ္သည္။

10. အေရးႀကီးသည့္ ေသြးအားနည္းေရာဂါ ရွိသူမ်ားသည္ ပံုမွန္ ေသြးလဲေပးရန္ လိုအပ္သည္။

11. ေသြးအားနည္းေရာဂါ ရွိသူသည္ ေနာက္ဆံုးတြင္ အေရးႀကီးသည့္ ေသြးအားနည္းေရာဂါ ရွိသူ ျဖစ္ႏိုင္သည္။

12. ေသြးအားနည္းေရာဂါသည္ ေသာက္ေဆးျဖင့္ ကုသႏိုင္သည္။

ျပင္းထန္ ေသြးအားနည္းေရာဂါသို႔ ဦးတည္သည့္ အေနအထား (စိတ္ပညာအရ ႀကိဳက္ျခင္း၊ မႀကိဳက္ျခင္းအား တိုင္းတာရန္ အတြက္ အေကာင္းျမင္ျခင္း - လံုး၀ သေဘာတူ = 5; သေဘာတူ = 4; မေသခ်ာ = 3; သေဘာမတူ = 2; လံုး၀ သေဘာမတူ = 1; ေျပာင္းျပ န္အားျဖင့္ အဆိုးျမင္ျခင္း)

ပထမဦးစြာ ျပင္းထန္ ေသြးအားနည္းေရာဂါရွိသည့္ ကေလးသည္ ေရာဂါ ခံစားရၿပီး မၾကာခဏ ဖ်ားနာႏိုင္ေၾကာင္း ရွင္းျပပါ။

13. ေသြးအားနည္းေရာဂါသည္ ေယဘုယ် လူဦးေရတြင္ တားဆီးသင့္သည္။

14. ေသြးအားနည္းေရာဂါရွိသည့္ ကေလးသည္ ၀န္ထုပ္၀န္ပိုး ျဖစ္သည္။

15. ေသြးအားနည္းေရာဂါရွိေသာ ကေလးသည္ ေကာင္းခ်ီးေပးခံရျခင္း ျဖစ္သည္။ (ကံေကာင္းတယ္လို႔ မွတ္ယူျခင္းကုိ ဆိုလုိ)

16. ေသြးအားနည္းေရာဂါသည္ ကေလးရွိလွ်င္ သိလိုပါသည္။

17. ေသြးအားနည္းေရာဂါ စစ္ေဆးမႈ ရႏိုင္လွ်င္ ကိုယ္၀န္ေဆာင္စဥ္အတြင္း စစ္ေဆးမႈ ရလိုပါသည္။

18. ေသြးအားနည္းေရာဂါ စစ္ေဆးမႈ ရႏိုင္လွ်င္ ကိုယ္၀န္ေဆာင္စဥ္အတြင္း အိမ္ေထာင္ဘက္အား စစ္ေဆးမႈ ရယူရန္ ဆႏၵရွိပါသည္။

19. ကၽြႏု္ပ္တို႔ ႏွစ္ဦးစလံုးတြင္ ေသြးအားနည္းေရာဂါရွိလွ်င္ အိမ္ေထာင္ဘက္ႏွင့္ ကေလး ဆက္လက္ ရယူလိုပါသည္။

20. ကၽြႏု္ပ္၏ ကေလးတြင္ ေသြးအားနည္းေရာဂါရွိခဲ့လွ်င္ သိလိုသည့္ ဆႏၵမရွိပါ။

21. ကိုယ္၀န္ေဆာင္ေနစဥ္ အေတာအတြင္း ေသြးအားနည္းေရာဂါ စစ္ျခင္းသည္ အသံုးမ၀င္ေပ။

22. ကၽြႏု္ပ္တို႔ ႏွစ္ ဦးစလံုးတြင္ ေသြးအားနည္းေရာဂါရွိလွ်င္ အိမ္ေထာင္ဘက္ႏွင့္ ေနာက္ထပ္ကေလး မလိုခ်င္ပါ။

23. ကေလးတြင္ ေသြးအားနည္းေရာဂါရွိခဲ့လွ်င္ ကိုယ္၀န္ဖ်က္ခ်လိုပါသည္။

24. ကိုယ္၀န္ဖ်က္ခ်ရန္ သေဘာမတူပါ။

25. ေမြးလာမည့္ ေသြးအားနည္းေရာဂါသည္ ကေလးငယ္အား မဖ်က္ခ်လိုပါ။

26. ေသြးအားနည္းေရာဂါသည္ ကေလးငယ္အား ေမြးဖြားၿပီး ခံစားေစျခင္းထက္ ကိုယ္၀န္ေဆာင္စဥ္ ဖ်က္ခ်ျခင္းက ပိုေကာင္းသည္။

လက္ေတြ႔လုပ္ေဆာင္ျခင္း (ရွိ / မရ

27. ယခင္က ေသြးအားနည္းေရာဂါ စစ္ေဆးခဲ့ဖူးပါသည္။

28. အိမ္ေထာင္ဘက္သည္ ယခင္က ေသြးအားနည္းေရာဂါ စစ္ေဆးခဲ့ဖူးပါသည္။

29. (ေမးခြန္းနံပါတ္ ၂၇ သို႔ ၂၈ အား “ရ ဟု ေျဖခဲ့လွ်င္) ကၽြႏု္ပ္ သို႔မဟုတ္ ကၽြႏု္ပ္၏ အိမ္ေထာင္ဘက္သည္ ကေလးအား ေသြးအားန

ည္းေရာဂါေၾကာင့္ ထိခိုက္မႈ႕ျဖစ္ျခင္းအား ရွာေဖြရန္ မီးမဖြားခင္ စစ္ေဆးခဲ့သည္။

30. (ေမးခြန္းနံပါတ္ ၂၉ အား “ရ ဟု ေျဖခဲ့လွ်င္) သင့္ကေလးတြင္ ေသြးအားနည္းေရာဂါရေွိ ၾကာင္း ျပသရန္ မိီးမဖြားခင္ စစ္ေဆးခဲ့ပါ

သလား -  စစ္ေဆးခဲ့ပါသည္  မစစ္ေဆးခဲ့ပါ

31. အနာဂတ္တြင္ ကေလး ၁ ေယာက္ သို႔မဟုတ္ ပိုယူဖို႔ အစီအစဥ္ ရွိပါသည္။

**Supplemental Figure 3D.** The Knowledge, Attitudes, and Practices (KAP) survey developed for the study of thalassemia in Khmer.

**ការវាស់ KAP:**

ចំណេះដឹង (បាទ/ចាស ,ទេ)

1. Thalassemia គឺជាជំងឺនៃឈាម។

2. Thalassemia គឺជាមូលហតុមួយដលបង្កឱ្យមានភាពស្លកសាំង។

3. Thalassemia គឺជាជំងឺកម្រមួយនៅអាស៊ី។

4. Thalassemia គឺជាជំងឺឆ្លងមានន័យថាអ្នកអាចឆ្លងវាបានពីអ្នកដទៃ។

5. Thalassemia គឺជាជម្ងឺមួយដែលបណ្តាលមកពីជម្ងឺដែលឪពុកម្តាយអាចចម្លងវាទៅកូន ៗ របស់ពួកគេ។

6. ថ្វីបើឪពុកមា្តយមិនមានជម្ងឺ Thalassemia ក៏ដោយក៏កូនរបស់ពួកគេនៅតែអាចកើតជំងឺបាន។

7. ប្រសិនបើឪពុកម្តាយពីរនាក់មានកូនដែលមានជំងឺ Thalassemia ពួកគេនឹងមានហានិភ័យក្នុងការមានកូនមួយផ្សេងទៀតដែលមានជំងឺ Thalassemia ។

8. ការធ្វើតេស្តឈាមអាចធ្វើបានដើម្បីពិនិត្យមើលថាតើនរណាម្នាក់មានជំងឺ Thalassemia ។

9. អ្នកជំងឺ Thalassemia ត្រូវការការចាក់បញ្ចូលឈាមឱ្យបានទៀងទាត់។

10. មនុស្សដែលមានជំងឺ Thalassemia តម្រូវការការចាក់បញ្ចូលឈាមជាទៀងទាត់។

11. អ្នកជម្ងឺ Thalassemia អាចនឹងវិវត្តទៅជាជំងឺ Thalassemia ។

12. Thalassemia អាចត្រូវបានព្យាបាលដោយថ្នាំគ្រាប់។

ឥរិយាបថឆ្ពោះទៅរក Thalassemia ធ្ងន់ធ្ងរ (មាត្រដ្ឋាន Likert សម្រាប់ធាតុវិជ្ជមាន: យល់ស្របទាំងស្រុង = 5 យល់ព្រម = 4 មិនប្រាកដ = 3 មិនយល់ស្រប = 2 មិនយល់ស្របទាំងស្រុង = 1 ផ្ទុយទៅវិញសម្រាប់ធាតុអវិជ្ជមាន)

ដំបូងសូមពន្យល់ថាកុមារដែលមានជំងឺ Thalassemia ធ្ងន់ធ្ងរទំនងជាឈឺញឹកញាប់និងមានជំងឺ។

13. Thalassemia គួរតែត្រូវបានការពារដោយប្រជាជនទូទៅ។

14. ការមានកូនដែលមានជំងឺ Thalassemia គឺជាបន្ទុកមួយ។

15. ការមានកូនដែលមានជំងឺ Thalassemia គឺជាពរជ័យមួយ (ឧ។ អាចចាត់ទុកថាមានសំណាង) ។

16. ខ្ញុំចង់ដឹងបើកូនខ្ញុំមានជម្ងឺ Thalassemia ។

17. ខ្ញុំនឹងត្រូវធ្វើតេស្តក្នុងអំឡុងពេលមានផ្ទៃពោះប្រសិនបើការធ្វើតេស្ត Thalassemia អាចបាន។

18. ខ្ញុំចង់អោយដៃគូរបស់ខ្ញុំធ្វើតេស្តក្នុងអំឡុងពេលមានផ្ទៃពោះប្រសិនបើការធ្វើតេស្ត Thalassemia អាចបាន។

19. ខ្ញុំនឹងបន្តមានកូនជាមួយដៃគូរបស់ខ្ញុំបើយើងទាំងពីរនាក់មានផ្ទុក Thalassemia ។

20. ខ្ញុំមិនចង់ដឹងថាតើកូនរបស់ខ្ញុំមានជំងឺ Thalassemia ។

21. ការធ្វើតេស្តរកជំងឺ Thalassemia មិនមានប្រយោជន៍សម្រាប់ខ្ញុំក្នុងអំឡុងពេលមានផ្ទៃពោះ។

22. ខ្ញុំមិនមានកូនច្រើនជាមួយដៃគូរបស់ខ្ញុំទេប្រសិនបើយើងមានផ្ទុក Thalassemia ។

23. ខ្ញុំនឹងបញ្ចប់ការមានផ្ទៃពោះបើទារកមានជំងឺ Thalassemia ។

24. ខ្ញុំមិនយល់ស្របនឹងការបញ្ចប់ការមានផ្ទៃពោះទេ។

25. ខ្ញុំនឹងមិនបងា្ករទារក Thalassemia ពីការកើត។

26. វាជាការល្អប្រសើរក្នុងការបញ្ចប់ការមានផ្ទៃពោះជាមួយទារក Thalassemia ជាជាងឱ្យកុមារត្រូវបានកើតនិងទទួលរង។

ការអនុវត្តន៍ (បាទ/ចាស ,ទេ)

27. ខ្ញុំត្រូវបានធ្វើតេស្តឈាមមុន។

28. ដៃគូរបស់ខ្ញុំត្រូវបានធ្វើតេស្តឈាមមុនពេល។

29. (ប្រសិនបើ បាទ/ចាស ,ទេ ចំពោះសំណួរទី 27 ឬ 28) ខ្ញុំឬដៃគូរបស់ខ្ញុំមានរោគវិនិច្ឆ័យមុនសម្រាល (PND) ដើម្បីរកឱ្យឃើញថាទារកត្រូវបានប៉ះពាល់ដោយជំងឺ Thalassemia.

30. (ប្រសិនបើ បាទ/ចាស ,ទេ នៅសំណួរទី 29) តើ PND បង្ហាញថាកូនរបស់អ្នកមានជំងឺរលាកថ្លើមទេ?

□បាទ/ចាសបា □ទេ

31. ខ្ញុំមានគំរោងនឹងមានកូន 1 រឺច្រើននាក់នាពេលអនាគត។

**References:**

1. Fucharoen S, Weatherall DJ. Progress Toward the Control and Management of the Thalassemias. Hematol Oncol Clin North Am. 2016;30(2):359-71.

2. Ismail JB. Thalassaemia and haemoglobinopathies in Brunei Darussalam. Med J Malaysia. 1992;47(2):98-102.

3. Savongsy O, Fucharoen S, Fucharoen G, Sanchaisuriya K, Sae-Ung N. Thalassemia and hemoglobinopathies in pregnant Lao women: carrier screening, prevalence and molecular basis. Ann Hematol. 2008;87(8):647-54.

4. Wongprachum K, Sanchaisuriya K, Dethvongphanh M, Norcharoen B, Htalongsengchan B, Vidamaly V, et al. Molecular Heterogeneity of Thalassemia among Pregnant Laotian Women. Acta Haematol. 2016;135(2):65-9.

5. Viprakasit V, Lee-Lee C, Chong QT, Lin KH, Khuhapinant A. Iron chelation therapy in the management of thalassemia: the Asian perspectives. Int J Hematol. 2009;90(4):435-45.

6. Viprakasit V, Ekwattanakit S, Riolueang S, Chalaow N, Fisher C, Lower K, et al. Mutations in Kruppel-like factor 1 cause transfusion-dependent hemolytic anemia and persistence of embryonic globin gene expression. Blood. 2014;123(10):1586-95.

7. Riolueang S, Ekwattanakit S, Korchuenjit J, Korchuenjit W, Tanyut P, Clark B, et al. Identification of a novel alpha0-thalasssemia (Siam; --SIAM), a rare 239 kb deletion of the alpha-globin cluster and the molecular application from routine DNA testing to prenatal diagnosis (PND) and preimplantation genetic diagnosis (PGD). 24th National Thalassemia Conference; August 21-23, 2019; Chiang Rai, Thailand.
